# Snakebite associated thrombotic microangiopathy: a systematic review of clinical features, outcomes, and evidence for interventions including plasmapheresis

**DOI:** 10.1371/journal.pntd.0008936

**Published:** 2020-12-08

**Authors:** Tina Noutsos, Bart J. Currie, Rachel A. Lek, Geoffrey K. Isbister

**Affiliations:** 1 Menzies School of Health Research, Charles Darwin University, Darwin, Northern Territory, Australia; 2 Flinders Health and Medical Research Institute, Flinders University, Australia; 3 Division of Medicine, Royal Darwin Hospital, Darwin, Northern Territory, Australia; 4 Clinical Toxicology Research Group, University of Newcastle, Newcastle, New South Wales, Australia; Universidad de Costa Rica, COSTA RICA

## Abstract

Snakebite is a neglected tropical disease with significant morbidity and mortality. Thrombotic microangiopathy (TMA) is an important but poorly understood complication of snakebite associated with acute kidney injury (AKI). Numerous treatments have been attempted based on limited evidence. We conducted a systematic review of TMA following snakebite using a pre-determined case definition of blood film red cell schistocytes or histologically diagnosed TMA. The search strategy included major electronic databases and grey literature. We present a descriptive synthesis for the outcomes of AKI, dialysis free survival (DFS), other end-organ damage, overall survival, and interventions with antivenom and therapeutic plasmapheresis (TPE). This study was prospectively registered with PROSPERO (CRD42019121436). Seventy-two studies reporting 351 cases were included, predominantly small observational studies. Heterogeneity for study selection, design, reporting and outcomes were observed. The commonest envenoming species were hump-nosed vipers (*Hypnale spp*.), Russell’s viper (*Daboia russelii*) and Australian brown snakes (*Pseudechis spp*.). The prevalence of TMA was at least 5.4% in proven and probable *Hypnale* bites, and 10–15% of Australian elapid envenomings, AKI occurred in 94% (293/312) of TMA cases, excluding case reports. The majority of cases with AKI required dialysis. Included prospective and retrospective cohort studies reporting interventions and renal outcomes showed no evidence for benefit from antivenom or TPE with respect to DFS in dialysis dependant AKI. The Grading of Recommendations, Assessment, Development and Evaluations (GRADE) assessment for quality of accumulated evidence for interventions was low. The major complication of TMA following snakebite is AKI. AKI improves in most cases. We found no evidence to support benefit from antivenom in snakebite associated TMA, but antivenom remains the standard of care for snake envenoming. There was no evidence for benefit of TPE in snakebite associated TMA, so TPE cannot be recommended. The quality of accumulated evidence was low, highlighting a need for high quality larger studies.

## Author summary

Thrombotic microangiopathy (TMA) is an important complication of snakebite. It occurs in a subset of patients envenomed by snake species associated with blood clotting abnormalities. TMA is a disorder marked by the formation of blood clots and blood vessel wall damage in the micro-circulation, and it carries a risk of organ damage and failure. Prior to our systematic review, evidence predominantly consisted of case reports and case series studies, and one landmark literature review on TMA following snakebite. Recent studies increasingly reported the use of resource intense treatment with therapeutic plasmapheresis (TPE), with limited supporting evidence of benefit. Our systematic review is the first to collate the worldwide experience of TMA in snakebite. We found TMA reported in at least 5.4% of *Hypnale* bites in Sri Lanka, and 10–15% of Australian elapid envenomings. The main complication was kidney failure. The kidney failure improved in most cases, irrespective of treatment with either antivenom or TPE. The major limitation of our review was the quality of included studies. Whilst antivenom remains the standard of care for snake envenoming irrespective of any evidence for specific benefit in snakebite associated TMA, our systematic review raises questions about whether the use of TPE represents a wise use of resources. There is a high resourcing cost of TPE, potential risks of the treatment, and lack of evidence for benefit. As a neglected tropical disease, it is imperative that treatment strategies for snakebite make efficient use of limited resources. Follow up studies, including good quality prospective cohort or interventional studies, would be best placed to confirm our findings.

## Introduction

Snake envenoming is a significant and neglected global public health issue causing multiple potentially life-threatening toxin-mediated clinical syndromes. Global estimates from the World Health Organisation (WHO) estimate 2.7 million snakebites and 81,000 to 138,000 deaths per annum globally attributable to snakebite [1]. Early access to medical care is imperative, and antivenom is the standard of care for envenomed patients. Snakebite is classified as a category A neglected tropical disease by the WHO [1]. Neglected tropical diseases are predominantly communicable diseases prevalent in tropical and subtropical regions. They affect over one billion people in resource-limited settings, carrying significant economic cost for low-middle income countries. In the context of snake envenoming, this may mean limited access to antivenom, blood products, specialised hospital and intensive care, and ventilators. The WHO recently set a global target for a 50% reduction in snakebite associated mortality and morbidity by 2030 [1].

Hemotoxic clinical toxin syndromes carry a significant risk of haemorrhage and death. Snake venoms have toxins which can act as anti-coagulant toxins which inhibit the clotting cascade, or as pro-coagulant toxins which activate the clotting cascade and consume clotting factors [2]. The consumption coagulopathy is commonly referred to as a venom induced consumption coagulopathy (VICC). VICC is marked by prolonged clotting times, and clotting factor deficiencies (i.e. hypofibrinogenaemia, low factor V, low factor VIII) and an elevated D-dimer [2–5]. VICC has a rapid onset and resolves with neutralisation or inactivation of the toxins and synthesis of new clotting factors [3,6,7].

A subset of snake envenomings with VICC develop thrombotic microangiopathy (TMA); a different, poorly understood haemotoxic syndrome [6,8–17]. The pathological hallmarks of TMA include small vessel micro-thrombosis and endothelial damage [18–20]. A mechanical red cell fragmentation known as microangiopathic haemolytic anaemia (MAHA) ensues, seen as circulating red cell fragments (schistocytes) in the blood [18,21]. Diagnosis of TMA is established by either thrombocytopenia with MAHA; or less commonly tissue biopsy [18,20]. The main risk in TMA is vaso-occlusive organ damage [18]. In TMA following snakebite the major end organ injury appears to be renal [6,11]. Dialysis is the mainstay of therapy for acute kidney injury (AKI) in snakebite. Further understanding of TMA following snakebite has been limited by available evidence, predominantly small and observational studies, many using varied and ill-defined nomenclature regarding TMA and VICC [11].

TMA following snakebite has been compared to other TMA conditions, including thrombotic thrombocytopenic purpura (TTP) and haemolytic uraemic syndrome (HUS) [11,22]. TTP results from acquired or inherited deficiency in a disintegrin and metalloproteinase with a thrombospondin type 1 motif (ADAMTS-13). TTP has a high fatality rate, with patient outcomes including survival dramatically improved by TPE with fresh frozen plasma (FFP) volume replacement [18,19,21]. Some studies have proposed therapeutic plasmapheresis (TPE) as an effective treatment for the acute kidney injury (AKI) of TMA following snakebite [23–25]. Associations between TMA following snakebite and HUS have arisen primarily given the apparent renal predominant end organ injury of both disorders [25–27]. Whilst TPE is commonly used during the initial presentation of HUS, it is usually unsuccessful. HUS is usually toxin mediated secondary to enterohaemorrhagic E. Coli diarrhoea, or complement mediated and associated with underlying genetic mutations of complement genes [18,20]. Eculizumab, a monoclonal antibody which targets complement C5, is now considered first line therapy for complement mediated HUS [18–20]. Any association between TMA following snakebite and either TTP or HUS with respect to pathophysiology, long term outcomes or best treatment, has not been established.

We performed a systematic review of TMA following snakebite, using a prespecified case definition. A descriptive synthesis of baseline characteristics; clinical presentation; outcomes of AKI, dialysis free survival (DFS), other end organ damage and overall survival; and evidence for or against intervention with TPE is presented.

## Methods

### Search strategy and selection criteria

The protocol for this systematic review has been previously published [28] and registered with PROSPERO (CRD42019121436). We performed the systematic review according to the Preferred Items for Systematic review and Meta-Analysis (PRISMA) checklist (S1 Table) [29,30].

Database searches included Pubmed, Medline via EBSCO, the Cochrane library, and grey literature searches of Grey Matters checklist, Google Scholar, opengrey.eu, grelit.org, GreyNet, Grey Literature Report, and BIOSIS Previews. Our last search date was the 16^th^ March 2020. Searches were limited to human studies. Search terms included snakes, snakebites, venoms, thrombotic microangiopathies, thrombosis, erythrocytes, schistocytes, red cell fragments, haemolysis, kidney disease and multiple organ failure. MeSH terms were used when possible, or alternatively free text words via Boolean search (S2 Table).

Inclusion criteria for TMA following snakebite cases were human studies on suspected or confirmed snakebite together with definite features of TMA. Definite features of TMA were defined a priori, as either explicitly reported blood film red cell fragmentation (schistocytes); or histological findings of TMA evidenced by vascular small vessel micro-thrombosis or wall injury (S1 Text) [18–20].

All studies reporting original data were eligible for inclusion, including published and unpublished studies, reports, conference abstracts, dissertations and conference papers. There were no exclusions with respect to patient age, age of study or minimum follow up period. Exclusion criteria included review studies not reporting original data, animal or in-vitro studies.

The first author independently screened retrieved abstracts for relevance. Two independent reviewers then screened relevant full text articles for eligibility. Disagreements were resolved by discussion and consensus. The bibliographies of full reviewed journal articles were manually searched for potentially relevant publications.

### Data analysis

Data extraction was conducted by two independent reviewers. We contacted 32 corresponding authors for further information when email was available. Seven responded and provided numerical data not published in the original paper. Where publications with the same author contained partly or completely duplicated cases, data were merged.

For study design classification, case series and cohort studies were differentiated as described by Mathes et al [31], with cohort studies clearly using exposure based sampling, longitudinal follow up over time for the occurrence of outcomes, reporting data which enabled effect measures for a risk of an outcome, with the temporality between exposure and outcome well defined.

Where possible data were converted to SI units and unified. Bleeding was classified as minor or major as per the International Society of Thrombosis and Haemostasis guideline for non-surgical patients [32]. AKI was classified according to dialysis requirement, and renal recovery outcomes by DFS and chronic kidney disease (CKD) stage where reported data allowed. End stage kidney disease (ESKD) was defined as dialysis dependant stage 5 CKD. VICC was categorised where possible by partial or complete VICC as previously described [28]. If no coagulation studies were performed within the first 48 hours post bite, VICC was recorded as unable to be determined (S3 Table).

Two reviewers independently assessed risk of bias for included studies using the framework of Murad et al (S4 Table) [33]. The accumulated level of evidence was assessed by the Grading of Recommendations, Assessment, Development and Evaluations (GRADE) framework [34]. Agreement between the two independent reviewers for study eligibility and data extraction was assessed by Cohen’s Kappa coefficient.

A descriptive synthesis of findings was performed. It was not appropriate to quantitatively pool synthesised outcome results in a meta-analysis due to heterogeneity in cases, statistical reporting, study design and settings, definition of clinical outcomes and potential confounding between low-middle, and high-income countries. Findings were synthesised by stratification according to risk of selection bias for included studies. Studies with a low risk of selection bias were grouped and findings presented in a detailed descriptive synthesis. Studies with an unclear or high risk of selection bias were presented in a summary table of baseline characteristics, interventions and outcomes. Within studies, continuous data were expressed as median and interquartile range (IQR) except where otherwise specified. Nominal data were aggregated as frequencies or proportions for each study. Odds ratios were calculated for studies with a low risk of selection bias, for intervention with antivenom and the binary outcomes of AKI, dialysis dependant AKI, ESKD, DFS and overall survival; and for intervention with TPE, DFS.

Study screening and review was managed by Covidence systematic review software, Veritas Health Innovation, Melbourne, Australia www.covidence.org. Data were analysed by GraphPad Prism 8.2.0 for Windows, Graphpad Prism Software, La Jolla California USA www.graphpad.com.

## Results

**Fig 1:**
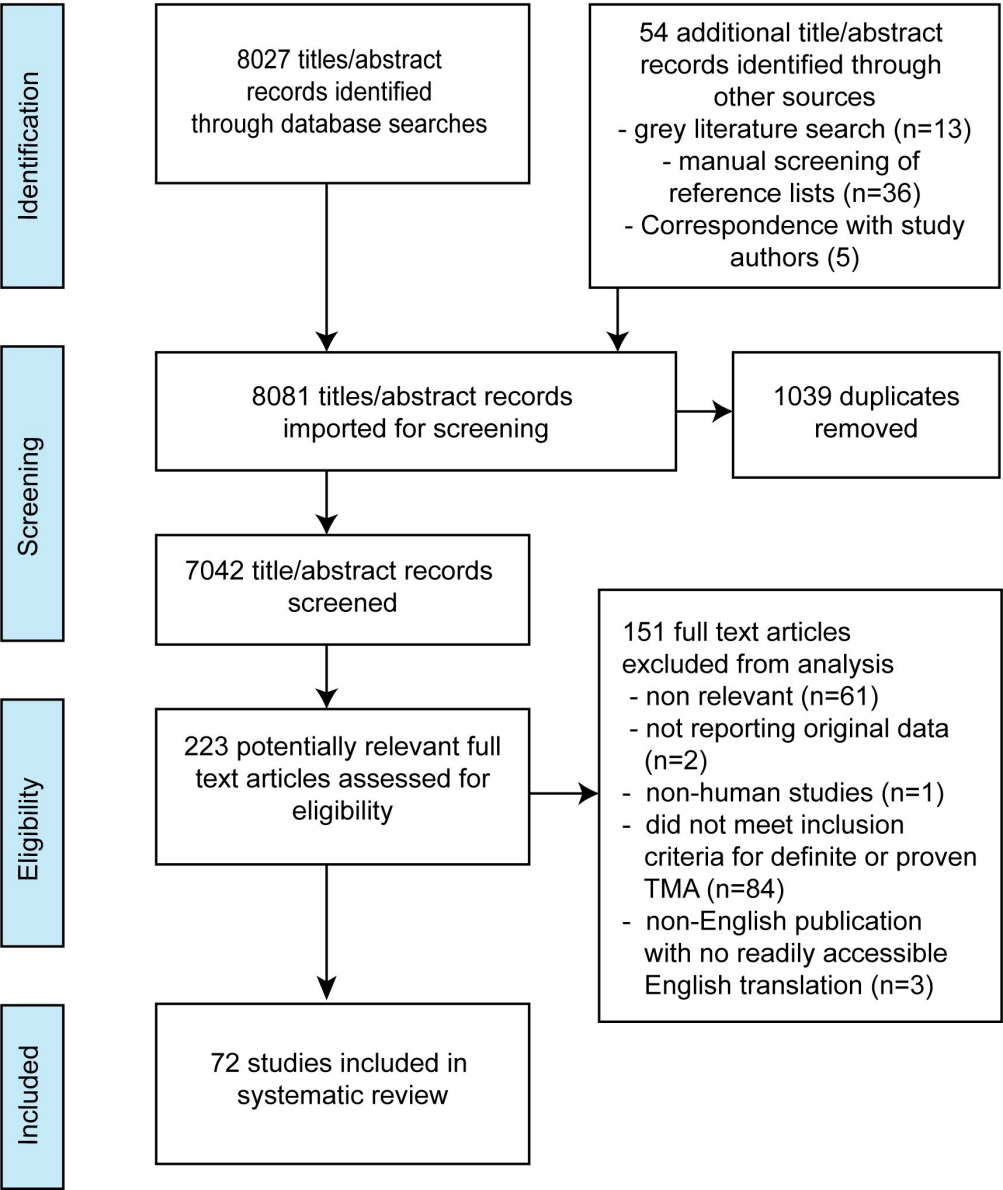
Study selection PRISMA study flow diagram derived from Moher D et al [29].

Seven-thousand-and-forty-two articles underwent abstract screening, with 223 full text reviews, and 72 studies selected (Fig 1). These included 31 single case reports [13,15,16,22–24,35–59], 31 case series [8,9,14,17,25–27,60–83], nine cohort studies [11,84–91], and one nested case control study (Table 1) [92]. Eighteen studies contained completely or partially duplicated cases which were merged (S5 Table) [9,11,14,23,26,41,60,66–68,75,76,81,84–86,88,92]. After merging, 351 unique cases fitting the inclusion criteria for TMA were included in our systematic review. Most cases resulted from snakebites in India (n = 164, 46.7%), Sri Lanka (n = 102, 29.1%) and Australia (n = 42, 12.0%). The envenoming snake was identified in 215 cases, of which 174 (80.1%) were vipers, 40 (18.6%) elapids and one (0.5%) colubrid. The commonest species were hump-nosed vipers (*Hypnale spp*.), Russell’s viper (*Daboia russelii*), Australian brown snakes (*Pseudechis spp*.), American pit-vipers *Bothrops spp*. and *Echis spp*. (Carpet and saw-scaled viper) (Table 1).

**Table 1:** Characteristics of included studies.

Baseline characteristic			
**Study design**			**n studies**
	Case reports		31
	Case series		31
	RCS and PCS		1
	Nested CCS		1
	Single centre RCS		3
	Single centre PCS		2
	Multicentre PCS		3
**Country**		**% cases**	n cases*
	Total	-	351
	India	46.7%	164
	Sri Lanka	29.1%	102
	Australia	12.0%	42
	Brazil	3.4%	12
	Burma	2.6%	9
	Nigeria	2.6%	9
	United States	1.1%	4
	Israel	0.6%	2
	United Kingdom	0.6%	2
	Thailand	0.3%	1
	Switzerland	0.3%	1
	Saudi Arabia	0.3%	1
	Seriname	0.3%	1
	Caribbean	0.3%	1
**Snake envenoming**		**% cases**	n cases*
	Total cases with snake identified	-	215
	Hump nosed viper (*Hypnale* spp)	25.6%	55
	Russell's viper (*Daboia russelii*)	22.8%	49
	Viper (species not reported)	17.7%	38
	Brown snake (*Pseudonaja* spp.)	12.6%	27
	*Bothrops* spp.	6.5%	14
	Saw scaled viper (*Echis carinatus*)†	4.7%	10
	Taipan (*Oxyuranus* spp.)	3.3%	7
	Tiger snake or tiger group (*Notechis* spp.)	1.4%	3
	*Demansia* spp.	1.4%	3
	*Echis coloratus*	0.9%	2
	Saharan horned viper (*Cerastes cerastes*)	0.9%	2
	Pigmy rattlesnake (*Sistrurus miliarius*)	0.5%	1
	Great Lakes bush viper (*Atheris nitschei*)	0.5%	1
	Lowland viper (*Proatheris superciliaris*)	0.5%	1
	Boomslang/South African green tree snake (*Dispholidus typus*)	0.5%	1
	Puff adder (*Bitis arietans*)	0.5%	1

*n cases after merging for duplicate cases.

†Subsequently renamed *E*. *ocellatus*, then *E*. *romani*. RCS: retrospective cohort study; PCS: prospective cohort study, CCS: case control study

**Fig 2:**
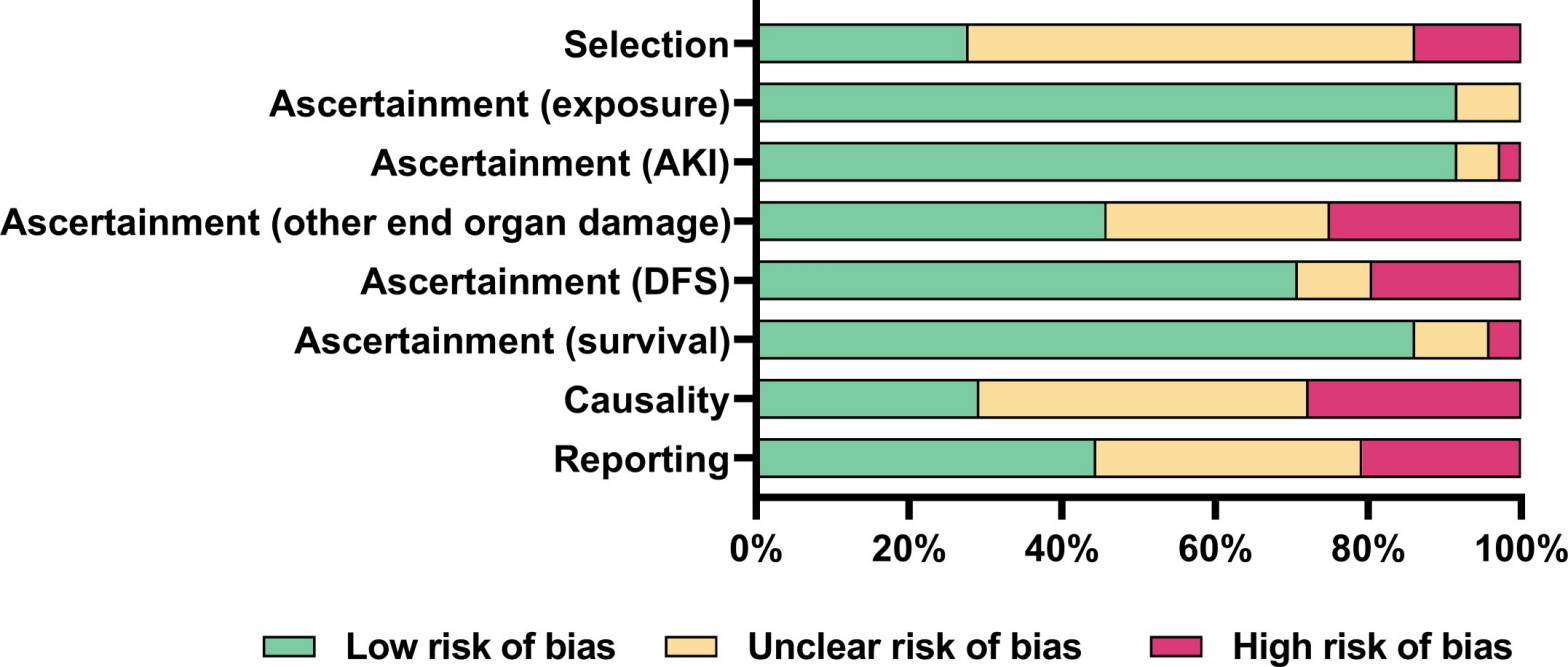
Risk of bias of included studies. Reviewer judgements about each risk of bias item presented as percentages across all included studies using the Murad et al risk of bias tool [33]. AKI: acute kidney injury; DFS: dialysis free survival.

Risk of bias was high or unclear in 72% of studies for selection methods; high or unclear in 71% of studies for causality; and high or unclear in 56% of studies for reporting (Fig 2 and S6 Table). Risk mostly pertained to whether selection methods allowed a true representation of the whole experience of the investigators; alternative causes for outcomes were appropriately ruled out; and cases were reported with sufficient detail for other practitioners to make inferences about their own practice. Risk of bias for ascertainment of outcomes was low in 92% of studies for exposure to snakebite envenoming; 92% of studies for AKI; 71% of studies for DFS; and 86% of studies for overall survival. Risk of bias for ascertainment of other end organ damage was unclear or high in 54% of studies, usually due to unclear or no reporting (Fig 2).

Twenty studies clearly represented the whole experience of study investigators, with defined recruitment and inclusion criteria (Tables 2 and S6). Studies of the Australian Snakebite Project reported TMA in 3.6% (31/856) of all Australian snakebites [81], 15% (6/40) of Australian taipan (*Oxyuranus* spp.) bites, and 10% (15/149) and 13% (4/32) of brown snake (*Pseudonaja* spp.) envenomings [11,85,86]. A Sri Lankan study found TMA in at least 5.4% (25/465) of suspected and proved *Hypnale* bites, using a TMA case definition of MAHA, thrombocytopenia and AKI [84]. Multiple studies reported histological findings of more highly selected cases of snakebite and AKI proceeding to renal biopsy and autopsy. Histological TMA changes in renal specimens were common (S7 Table) [61,62,69,74–76,79,87]. Most TMA cases occurred in adults, and males were moderately over-represented compared to females (Table 2).

**Table 2:** Characteristics of included studies representing whole experience of authors with low risk of selection bias.

Study	Study design	Study duration (years)	Country	Participant group/cohort and setting	TMA (n)	Snake species	Age	Sex M/F	Presenting features	Clinical end organ injury (n)
Acharya (1989)[61]	CS	15	India	Snakebite with AKI admitted to a single centre, 50 cases in total, of which 29 underwent renal biopsy or autopsy	≃ 11	Viperidae	NR	NR	NR. Histological study, TMA in ≃ 7 autopsies, ≃ 4 live renal biopsies	Renal (11)
Australian Snakebite Project*[11,81,85,86]	Multi centre PCS	13 (2003–2016)	Australia	Multiple PCS from the Australian Snakebite Project, containing partly duplicated cases. Allen et al [11,85] reported an 8 year PCS cohort of 149 definite brown snakebites presenting to hospital, of which 136 had systemic envenoming. All systemic envenomed cases had VICC, 15 (10%) of which developed TMA. Johnston et al [86] reported on 40 Australian Taipan snakebites, of which 33 had neurotoxicity, 16 complete VICC, 15 partial VICC, 13 AKI (3 of which required dialysis), 11 myotoxicity, and 6 (15%) TMA. Noutsos et al [81] reported 856 total snakebites, of which 319 had VICC, and 31 had TMA (3.6% of total bite and 9.7% of VICC cases)	34	Brown snake (*Pseudonaja* spp. (23), Taipan (*Oxyuranus* spp.) (6), Tiger snake or tiger group (*Nochetis* spp.) (3), unknown (2)	47 (35–59) (median, IQR) (n = 31), NR (n = 3)	23M/8F	Schistocytes (34). VICC (34)–complete (22), partial (9), category NR (3). Anaemia and thrombocytopenia (31), NR (3): Hb nadir 83 (65–107) (median, IQR), platelet nadir 31 (17–69) (median, IQR) (n-31)	Renal (29), no renal injury (4), renal injury NR (1). Pancreatitis and NSTEMI (1)‡
Amaral[62] (1985)	CS	10	Brazil	Admissions to intensive care unit with AKI due to Bothrops snakebite, 22 cases, of which 7 were biopsied and showed renal cortical necrosis, TMA in 6 (2 autopsies, 4 renal biopsies)	6	*Bothrops jararaca* (4), *Bothrops jararacussu* (1), *Bothrops* spp. (1)	51–66	3/3	Anaemia (6)	Renal (6). Other organ damage NR
Chugh (1984, 1975)* [9,66]	CS	16 (1964–1980)	India	157 snakebites, of which 45 developed AKI, of which 35 had histology performed	≥5	Russell’s viper (*D*. *russelii*) (1), viperidae (1), NR (3)	20, 26, NR (n = 3)	1F/1M, NR (n = 3)	Bleeding (2), bleeding NR (3). Partial VICC (2), coagulation studies NR (4). Schistocytes (5). Anaemia (2), Hb nadir NR (4). Thrombocytopenia (2), platelet nadir NR (4).	Renal (5). Other organ damage NR
Date (1986)[26]	CS	8	India	Snakebite and acute renal failure, 24 patients, of which at least 22 definite TMA	≥22	*D*. *russelii*	23–50 (11), NR (11)	6/5 NR (n = 11)	Bleeding (11) of which at least 1 major GI bleed, no bleeding (1), bleeding NR (10). Schistocytes (22). Anaemia and thrombocytopenia (16). Partial VICC (11), coagulation studies NR (11)	Renal (22), other organ damage NR
Gupta (1988)[69]	CS	4 (1978–1982)	India	Snakebite admitted to hospital, 121 snakebites, 15 with oliguric AKI, 7 of which had histology performed, with 1 (autopsy) TMA	1	NR	NR	NR	NR	Renal (1)
Merchant (1989)[74]	CS	8 (1977–1985)	India	Snakebite and AKI, 50 total cases–*D*. *Russelii* (13), *Echis*. *Carinatus* (10), sea snake (1), unidentified (24). Of these 29 had renal histology performed (15 of these autopsies), at least 11/29 TMA, total number unclear	≥11	NR	NR	NR	NR	Renal (11). Other organ damage NR
Milani Junior (1997)[87]	Single centre RCS/ PCS	20	Brazil	Proven Jararacucu snakebites presenting to 2 hospitals, 29 cases total, of which 14 had coagulopathy, 4 AKI, 3 deaths. 2 cases had ATN, cerebral oedema, rhabdomyolysis. 2 definite TMA cases	2	*B*. *jararacussu*	35, 65	2M	Minor bleed (1), no bleed (1). Anaemia and thrombocytopenia (1), NR (1). Complete VICC (1), partial VICC (1).	Renal (2). Bowel and meningeal clinically and at autopsy (1)
Mittal (1994, (1986)[75, 76]*	CS	23 (1971–1993)	India	Renal histology specimens from cases with snakebite and AKI proceeding to renal biopsy or autopsy, 41 total cases, of which ≃ 25 TMA (14 autopsy, 11 live biopsy), subject to reporting and interpretation of histology	≃ 25	Viperidae (*D*. *Russelii*, *Echis*. *Carinatus*)	NR	NR	At least partial VICC (11), coagulation studies NR (14).	Renal (25). Other organ damage NR
Mohan (2019) [23,88]*	Single centre RCS	3	India	Snakebites admitted to single centre, 331 cases total, with 17 excluded due to insufficient case data. Of remaining 314 cases, 202 were haemotoxic, of which 36 (19%) were TMA (using case definition of TMA of MAHA, thrombocytopenia and AKI), and an additional 11 (5%) were MAHA with schistocytes, thrombocytopenia and no AKI.	47	NR	49.1 +/- 13.43 (mean, SD) for 36 with AKI	25/11 for 36 with AKI	VICC (26), coagulopathy not classifiable (1), no coagulopathy (9)†, coagulation studies NR (11). Schistocytes (47). Anaemia and thrombocytopenia (47).	Renal (36), no renal injury (11). Possible other organ damage‡ including multiorgan dysfunction syndrome (3), ARDS(3), myocarditis (2), seizure (2), MI (2)
Namal (2019)[84]	Single centre PCS	4 (2014–2018)	Sri Lanka	Proven and probable hump nosed viper bites presenting to hospital. 465 hump nosed viper bites, 44 of which (9.5%) developed AKI, of which 23 (5%) proven and 21 (4.5%) probable hump nosed viper snakebite cases not able to be definitively speciated. Of proven cases, 17% (4) progressed to CKD, and 12 (52%) had TMA defined by thrombocytopenia, MAHA and AKI. Of probable cases, 17 had MAHA defined by 3 schistocytes per high power field on microscopy of blood films, of which 13 (62%) had TMA defined by authors as thrombocytopenia, MAHA and AKI	29	*Hypnale*. *Hypnale* (12), *Hypnale* spp (17)	NR	NR	No coagulopathy (10), coagulopathy not classifiable (2), coagulation studies NR (17). Schistocytes (29), MAHA (29), thrombocytopenia (25).	Renal (29). Neurological TTP-like presentation (1)
Rao (2019)[89]	Single centre RCS	6 (2012 to 2017)	India	Patients over 18 years admitted with definitive snakebite and AKI. Patients with a known history of CKD were excluded. 103 total cases, of which 19 (18.5%) had TMA defined by MAHA with >1% schistocytes on blood film microscopy, a platelet nadir of <100x10^9^/L and AKI in the absence of alternative causes (eg. sepsis)	19	NR	52.7 +/- 11.14 (mean, SD)	13M/6F	VICC defined as WBCT >20 min and/or both prolonged APTT/INR (4). Bleeding (3), no bleeding (16). Schistocytes (19). Anaemia and thrombocytopenia (19).	Renal (19). Other organ damage‡: myocarditis (2), ARDS (3)
Than-Than (1989)[79]	CS	2 (1983–1985)	Burma	All patients admitted to single centre hospital with snakebite over November to December rice harvest seasons, 199 total cases, 10 fatalities, 3 which consented to autopsy, 2 of which were TMA.	2	*D*. *russelii*	17,19	2M	Minor bleeding (1), no bleeding (1), complete VICC (1), coagulation studies NR (1),	Renal (2). Other organ damage‡: pituitary and lung TMA on autopsy (2), clinical organ function NR
Warrell (1977)[90]	Single centre RCS	3	Nigeria	Snakebites presenting to single centre, Total 204 cases, of which 181 bites *E*. *carinatus*. Serial blood films examined in 42 patients, of which 1 had schistocytes, and another 7 showed more mild changes of schistocytes and sphering, therefore 8/42 (19%) TMA cases	8	Saw scaled viper (*Echis carinatus*)	12 (1), others NR	1M, others NR	Schistocytes (8). Complete VICC (1), incoagulable blood (7). Bleeding, anaemia, and thrombocytopenia (1); others NR (7)	Renal (1), renal injury NR (7). Other organ damage NR
Wijewickra-ma (2020)[91]	Single centre PCS	3	Sri Lanka	Hospital admissions with AKI secondary to snakebite, 80 total cases, 59 included in analysis with complete data for first week post snakebite. Of 59 cases, 45 TMA (defined as MAHA with schistocytes, thrombocytopenia and AKI) and an additional 2 had MAHA with schistocytes and AKI without thrombocytopenia	47	10 *Daboia* spp, 19 *Hypnale* spp, 18 unidentified	Median (IQR):60 (56–66) (n = 18); 56 (47–68) (n = 18); 46 (39–59) (n = 9)	23M/22F, 2 NR	Schistocytes (47). Anaemia and thrombocytopenia (45), MAHA and no thrombocytopenia (2). VICC (15), no VICC (6), coagulation studies NR (26)	Renal (47). Other organ damage NR

*Studies merged due to duplicate cases.

†authors recommended caution with interpretation of cases with no coagulopathy due to delayed hospital presentation (3.6+/- 4.8 days) of these cases.

‡Attribution to TMA unclear. TMA: thrombotic microangiopathy; CS: case series; AKI: acute kidney injury; NR: not reported; PCS: prospective cohort study; VICC: venom induced consumption coagulopathy; WBCT: whole blood clotting time; Hb: haemoglobin; IQR: interquartile range; NSTEMI: non-ST elevation myocardial infarction; RCS: retrospective cohort study; MI: myocardial infarction; ARDS: acute respiratory distress syndrome; CKD: chronic kidney disease; ATN: acute tubular necrosis; MAHA: microangiopathic haemolytic anaemia

Thirteen studies with low risk of selection bias reported rates of coagulopathy (Table 2). Nine studies found coagulopathy in 100% of TMA cases [9,11,26,75,79,85–87,90]. The remaining studies reported coagulopathy in only a proportion of cases. However time to hospital presentation in these was typically prolonged, meaning VICC may have been missed [84,88,89,91]. Coagulation findings were heterogeneously reported. Some studies reported data allowing classification as VICC; others reported non- laboratory based tests including whole blood clotting times (WBCT). Bleeding was common, typically minor and mucocutaneous (Table 2). Major haemorrhage occurred in 2/15 (13%) of Australian brown snakebites with TMA, of which one was a fatal intracranial haemorrhage [85]; and 2/12 (17%) of Russell’s viper snakebites with TMA, both gastrointestinal, one of which was fatal [26,67]. Moderate thrombocytopenia and anaemia with elevated haemolysis markers were almost universally present (Table 2 and S1, S2 and S3 Figs). VICC typically occurred soon after the onset of envenoming, and changes of MAHA including haemoglobin and platelet nadir, and maximum LDH in subsequent days (S4, S5, S6 and S7 Figs).

AKI was present in 94% (n = 293) of a total 312 included cases, after removal of case reports, where AKI was reported (Tables 2 and S5). Among individual studies with a low risk of selection bias, a single centre retrospective cohort study from India found AKI in 36/47 (77%) of snakebite with TMA [88]. Australian Snakebite Project studies found AKI in 26/30 (87%) of snakebite with TMA cases [81]. Remaining studies with low risk of selection bias were not representative of AKI prevalence in snakebite associated TMA, owing to: not reporting AKI frequency; using AKI as an inclusion criterion for TMA cases; or recruiting highly selected cases from renal units or renal biopsy pathology services.

**Fig 3:**
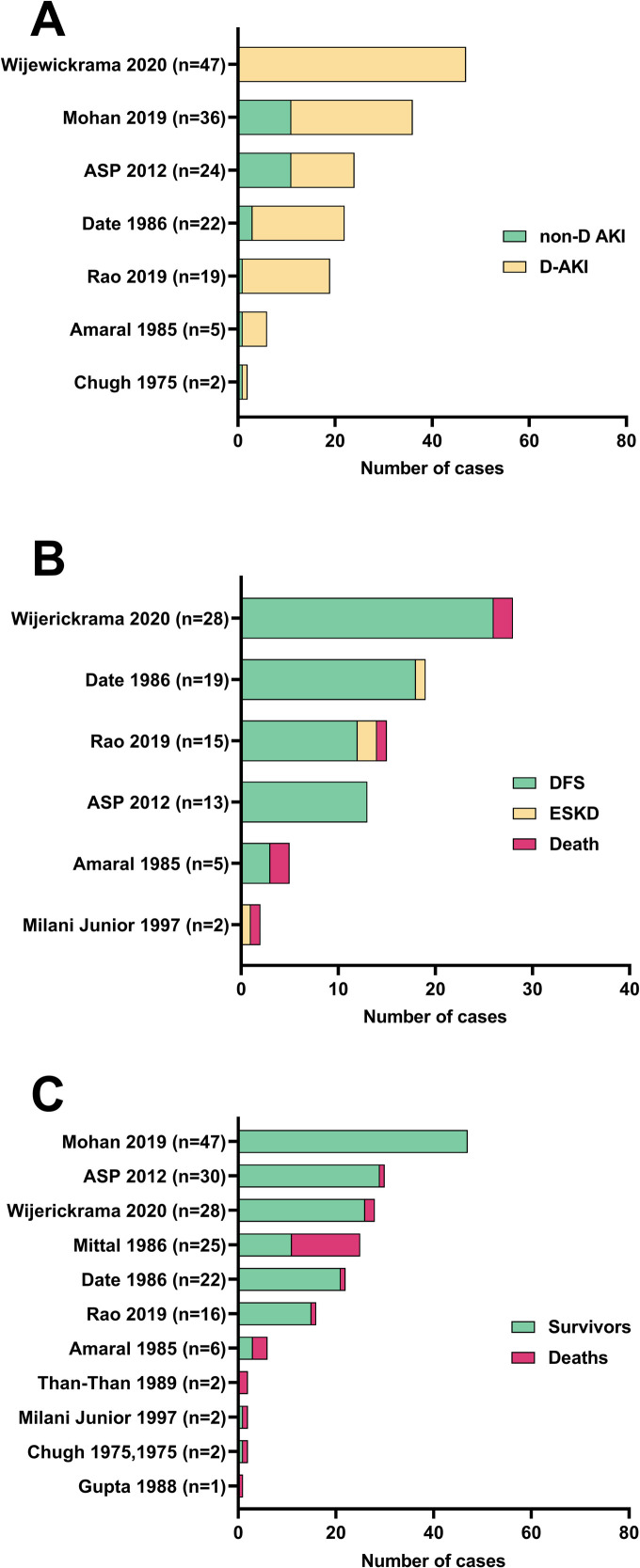
Study data for (A) dialysis dependant AKI (D-AKI) versus non-dialysis dependant AKI (non-D AKI); (B) dialysis free survival (DFS) for cases with dialysis dependant AKI; and (C) overall survival for all TMA cases for studies with low risk of selection bias. ESKD: End stage kidney disease.

Many TMA cases with AKI required dialysis support (Fig 3A). The largest study with a low risk of selection bias found dialysis dependence in all 47 Sri Lankan cases of TMA with AKI following viper bites [91]. However, this study was based in a specialist tertiary renal centre. Three Indian studies reported dialysis dependence in 25/36 (69%), 19/22 (86%) *D*. *russelii*, and 18/19 (95%) of snakebite associated TMA cases with AKI [26,88,89]. An Australian Snakebite Project study found dialysis dependence in 13/24 (54%) of snakebite associated TMA cases with AKI [11,81]. Other studies reported much smaller numbers of TMA cases with AKI, from highly selected intensive care units or renal pathology departments, most of which were dialysis dependant [9,62].

Most TMA cases with dialysis dependant AKI achieved DFS (Fig 3B). The three largest studies were from Indian and Sri Lankan tertiary hospital specialised renal centres. DFS for dialysis dependant AKI cases was 26/28 (93%) [91]; 18/19 (95%) *D*. *russelii* [26]; and 12/15 (80%) [89]; with cases lost to follow up 19/47 (40%) [91]; 0/19 [26]; and 3/18 (17%) [89] respectively. The Australian Snakebite Project found DFS in all 13 dialysis dependant AKI cases [11,81]. Two small studies of *Bothrops* envenomings in Brazil reported highly selected cases proceeding to renal biopsy [62,87]. Outcomes were poorer with DFS 3/5 (60%) and 0/2, respectively. Two recently published large cohort studies with low risk of selection bias did not explicitly report DFS outcomes for their patient cohorts [84,88]. Timing of dialysis dependence was reported in four studies with low risk of selection bias. In cases achieving DFS, dialysis dependence persisted for: 12 (7–24) days (median, IQR, n = 45) [91]; 18.0 +/- 8.1 sessions (mean, SD, n = 15) [89]; 13.0 (10.0–24.0) days (median, IQR, n = 9) [11,81]; and 2.0 (2.0–4.7) sessions (median, IQR, n = 8) [26,68].

Seven studies with a low risk of selection bias reported renal outcomes versus intervention with antivenom (Table 3). The largest study reporting data on antivenom intervention was by Mohan et al, reporting 36 snakebite associated TMA cases of which 28 received antivenom and 8 did not. AKI was present in all 36 cases. DFS was not specifically reported for cases, however survival was 100% in patients treated with and without antivenom [88]. A study by Namal et al reported 29 cases of snakebite associated TMA in *Hypnale* envenomings, for which no specific antivenom is available. All 29 cases had AKI, but specific outcomes of dialysis dependence and DFS were not reported [84]. The Australian Snakebite Project studies included 29 cases treated with antivenom, and one not treated with antivenom. In the antivenom group, AKI occurred in all cases, dialysis dependant AKI in 25/29 (86%), and DFS in the dialysis dependant AKI group was in 12/12 (100%). The one case not treated with antivenom developed a dialysis dependant AKI and achieved DFS. For all studies reporting antvenom intervention and renal outcomes, calculated odds ratios for the outcomes of AKI, dialysis dependant AKI, DFS and overall survival were non-significant (Table 3).

**Table 3:** Outcomes of AKI, DFS, death and overall survival for studies with a low risk of selection bias reporting intervention with antivenom.

Outcomes by study	AV n/N (%)	No AV n (%)	Calculated odds ratio	95% CI	p value
**Amaral 1985 [62]**	6	0			
AKI	6 (100%)	-	13.0	0.1–1680.9	0.30
D-AKI	5/6 (83%)	-	3.7	0.0–274.5	0.56
DFS	3/5 (60%)	-	0.7	0.0–49.7	0.88
ESKD	0/5	-	0.1	0.0–11.9	0.33
Death	2/5 (40%)	-	0.7	0.0–49.7	0.88
Overall survival	3/6 (50%)	-	1.0	0.0–66.1	1.00
**Australian Snakebite Project studies [11,14,41,81,85,86,92]**	29	1			
AKI	25/29 (86%)	1/1	1.9	0.1–54.1	0.71
D-AKI	12/25 (48%)	1/1	0.3	0.0–8.3	0.48
DFS	12/12	1/1	8.3	0.1–596.1	0.33
ESKD	0	0	1.0	0.0–255.6	1.00
Death	0	0	1.0	0.0–255.6	1.00
Overall survival	28/29 (97%)	1/1	6.3	0.2–231.1	0.31
**Chugh 1975 [9]**	2	0			
AKI	2 (100%)	-	5.0	0.0–711.9	0.52
D-AKI	1 (50%)	-	1.0	0.0–92.4	1.00
DFS	0	-	0.3	0.0–52.6	0.67
ESKD	0	-	0.3	0.0–52.6	0.67
Death	1	-	3.0	0.0–473.1	0.67
Overall survival	NR	-	-	-	-
**Mohan 2019 [88]**	28	8*			
AKI	28 (100%)	8 (100%)	3.35	0.1–182.0	0.55
D-AKI	NR	NR	-	-	-
DFS	NR	NR	-	-	-
ESKD	NR	NR	-	-	-
Death	NR	NR	-	-	-
Overall survival	28 (100%)	8 (100%)	3.5	0.1–182.0	0.55
**Milani Junior 1997 [87]**	2	0			
AKI	2	-	5.0	0.0–711.9	0.52
D-AKI	1/2 (50%)	-	1.0	0.-92.4	1.00
DFS	0	-	0.3	0.0–52.6	0.67
ESKD	1/1	-	3.0	0.0–473.1	0.67
**Outcome**	**AV n (%)**	**No AV n (%)**			
Death	0	-	0.3	0.0–52.7	0.33
Overall survival	1/1 (1 survival NR)	-	-	-	-
**Namal 2019 [84]**	0	29†			
AKI	-	29	0.0	0.0–2.1	0.10
D-AKI	-	NR	-	-	-
DFS	-	NR	-	-	-
ESKD	-	NR	-	-	-
Death	-	NR	-	-	-
Overall survival	-	NR	-	-	-
**Than-Than 1989 [79]**	2	0			
AKI	2	-	5.0	0.0–711.9	0.52
D-AKI	0	-	0.2	0.0–28.5	0.52
DFS	-	-	-	-	-
ESKD	-	-	-	-	-
Death	-	-	-	-	-
Overall survival	0	-	0.2	0.0–28.5	0.52

*Authors stated unreliable data on AV administration for those transferred from other centres.

†Presumed antivenom not given, study of *Hypnale* envenoming and specific antivenom not available. AV: antivenom; AKI: acute kidney injury; D-AKI: dialysis dependent AKI; DFS: dialysis free survival; ESKD: end stage kidney disease; NR: outcome not reported for AV intervention.

**Fig 4:**
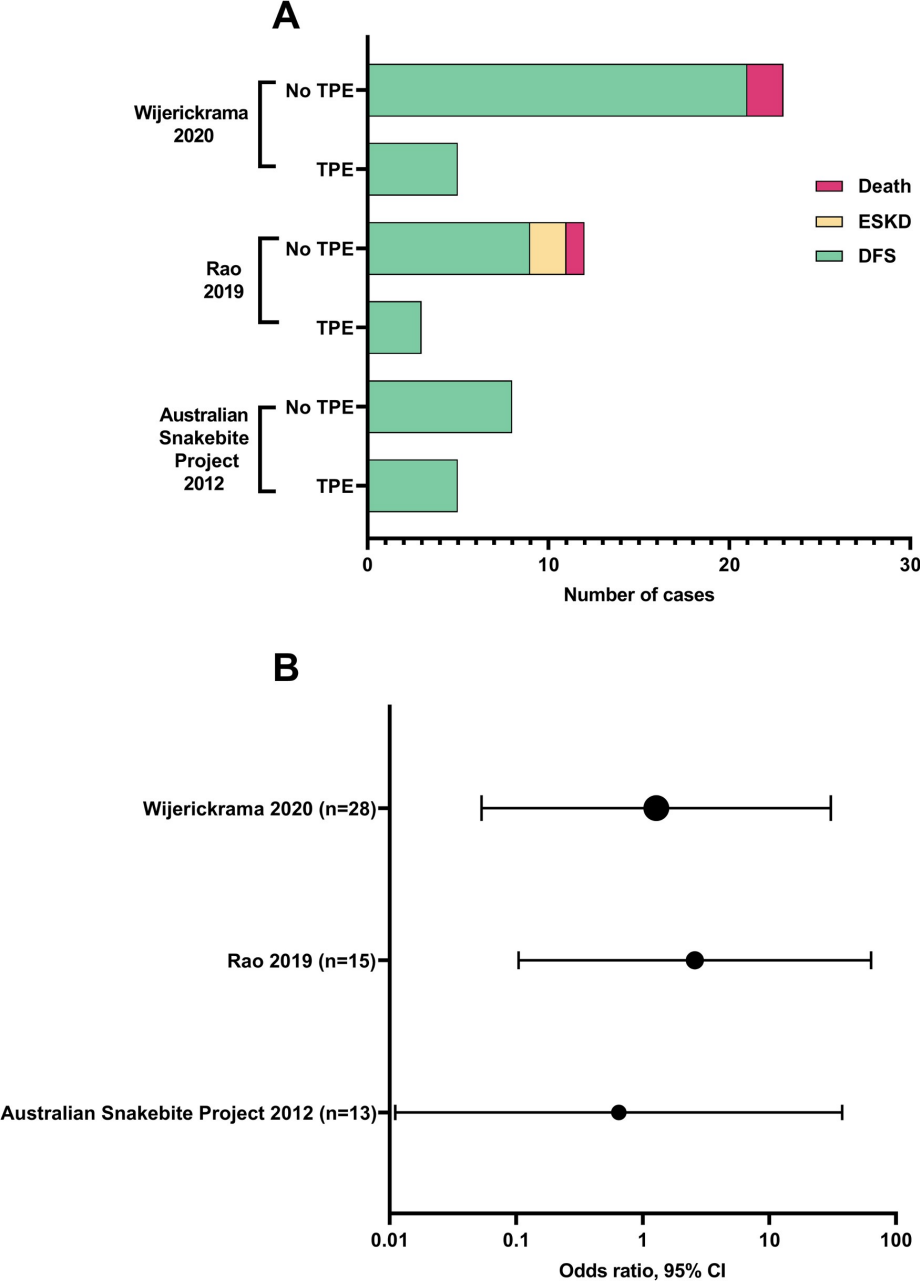
Dialysis free survival for plasmapheresis (TPE) versus non-TPE treated cases for studies with low risk of selection bias: (A) total number of cases; and (B) odds ratio of dialysis free survival (DFS) for TPE vs non-TPE. p>0.05 for all calculated odds ratios. ESKD: End stage kidney disease.

Three studies with a low risk of selection bias reported outcomes for DFS in cases with dialysis dependant AKI, versus intervention with TPE (Fig 4A). The largest was a Sri Lankan single centre prospective cohort study, reporting outcomes for intervention with TPE, FFP alone, and no TPE or FFP [91]. Dialysis dependence at time of discharge from hospital was higher in the TPE group (6/9, 67%), versus 7/17 (41%) for FFP alone; and 4/17 (24%) for no TPE or FFP. DFS at three months was 12/13 (92%) for no TPE or FFP; 9/10 (90%) for FFP alone; and 5/5 (100%) in the TPE treatment group. Patients who did not receive TPE had better renal function and less chronic kidney disease at three months compared to those given TPE. The authors found TPE was not associated with improved blood transfusion requirements, platelet count recovery, requirement for dialysis or hospital duration of stay. A second study from India found no significant difference in DFS for dialysis dependant AKI snakebite for patients who got TPE compared to those who did not [89]. The Australian Snakebite Project reported DFS in all patients, both TPE (n = 5) and non-TPE (n = 8) cases with dialysis dependant AKI [11,81]. We calculated odds ratios for DFS and TPE intervention for these studies and found a non-significant difference (p>0.05) in DFS between treatment groups (Fig 4B and S8 Table).

Two additional studies with low risk of selection bias reported patient groups with and without TPE intervention, without explicitly reporting DFS data [84,88]. Mohan et al reported 36 TMA cases with AKI from India, of which 25 required dialysis support. One received TPE, and 24/25 no TPE. Summary data for the whole 36 AKI cases was reported with 26/36 discharged without complication and 3/36 lost to follow up. DFS was not explicitly reported. The case treated with TPE achieved DFS, but had a persistently abnormal creatinine at six weeks [23,88]. Namal et al reported 44 *Hypnale* bite cases with AKI, of which 25 met our inclusion criteria for TMA. TPE was used in 7/44 cases; however, outcomes specific to the TMA group for dialysis dependence, DFS and overall survival for TPE versus no TPE intervention were not reported. Of all 44 cases with AKI, 30/44 (68%) required dialysis support, four were lost to follow up, 29/40 (72%) recovered, 9/40 (22%) developed CKD (of which at least one was treated with TPE), and three died [84].

Overall survival for all TMA cases was reported in 11 studies with low risk of selection bias (Fig 3C). The majority of cases survived, but survival outcomes between studies were heterogenous. Of these 11 studies, the largest four cohort studies reported survival of 100% (n = 47) in a retrospective cohort study from India [88], 97% (29/30) in the prospective Australian Snakebite Project studies, and 93% (26/28) and 94% (15/16) in two cohort studies of viper bites from Sri Lanka and India respectively [89,91]. One case series of more highly selected patients proceeding to renal biopsy or autopsy from India had poorer survival of 44% (11/25) [75]. Cause of death for all studies in which it was reported was most commonly shock, refractory hypotension, major haemorrhage, or organ failure.

Patient data for other organ damage except renal injury, was reported in six studies with a low risk of selection bias (Table 2). Other end organ damage was uncommon. Occasional cases of clinical multiorgan, cardiac, neurological, pituitary, lung, and bowel dysfunction were reported. However, attribution to TMA specifically as the cause was uncertain [81,84,87–89]. TMA findings were rarely reported in other organs at autopsy (Tables 2 and S7) [79,87].

Fifty-two studies eligible for inclusion in this systematic review had an unclear or high risk of selection bias, most case reports or small case series (Table 4). Most reported AKI which was dialysis dependant. Renal outcomes were heterogeneously or unclearly reported in many of these studies. The majority achieved DFS with or without TPE, however the studies were heterogenous in selection and outcomes, and did not clearly represent the entire experience of the study authors.

**Table 4:** Characteristics and main findings of included case series and case reports with high or unclear risk of selection bias.

Study*	Study design	TMA (n)	Country	Snake species	Age/Sex	Pathological evidence of TMA	Presenting features			Clinical end organ injury	Treatment	Last contact	Outcome
							↓Hb	↓plt	Coagulopathy				
Ahlstrom (1991)[35]	CR	1	USA	Pigmy rattlesnake (*Sistrurus miliarius*)	62 M	Schistocytes	Yes	Yes	Complete VICC	Dialysis requiring AKI, ? brain (cortical blindness and seizure day 3)	AV test dose only	15 d	DFS, creatinine 283μmol/L
Al Qahtani (2014)[36]	CR	1	Saudi Arabia	NR	38 M	Biopsy: kidney	Yes	Yes	Complete VICC	Dialysis requiring AKI	AV	2 mo	DFS
Aung (1978)[63]	CS	7	Burma	*D*. *russelii*	5M/2F	Biopsy kidney (4 autopsy, 3 live biopsy)	NR	NR	NR	AKI dialysis use NR	NR	NR	Death in 4 (autopsies), 3 live biopsies clinical outcomes NR
Basu (1977)[64]	CS	9	India	*D*. *Russellii*	NR	Biopsy kidney	NR	NR	NR	AKI dialysis use NR	NR	NR	NR
Benvenuti (2003)[37]	CR	1	Brazil	*Bothrops jararacussu*	36 F	Autopsy: heart, lung	NR	NR	NR. Major bleed–lung	NR	NR	45 min	Death from pulmonary haemorrhage
Bucaretchi (2019)[38]	CR	1	Brazil	*B*. *jararaca*	56 F	Schistocytes	Yes	Yes	WBCT >20 min	AKI not requiring dialysis	AV	10 mo	Survival, renal function normal
Chugh (1989)[65]	CS	2	India	*D*. *russelii* (1), Saw scaled viper (1)	17M	Biopsy kidney	Yes	Yes	Partial VICC, Major GI bleed	AKI requiring dialysis	AV	21 d	DFS, normal renal function
					58M	Biopsy kidney	Yes	Yes	Partial VICC, minor m/c bleed	AKI requiring dialysis	AV	6 mo	DFS at 4 weeks, ongoing Stage 5 CKD, later died at 6 months unknown cause
Cobcroft (1997)[24]	CR	1	Australia	Taipan (*Oxyuranus scutellatus*)	33 M	Schistocytes, autopsy: kidney, spleen, lung	Yes	Yes	Partial VICC	AKI, dialysis requirement NR	AV, TPE	3 w	Death from muscular weakness, cardiac arrest/hypoxic brain injury. Renal function not recovered
de Silva (2017)[16]	CR	1	Sri Lanka	Hump nosed viper (*Hypnale* spp.)	50 F	Schistocytes	Yes	Yes	NR	AKI requiring dialysis; ? cardiac (MI day 7)	-	33 d	Death day 33. Some renal recovery: ceased dialysis day 9. Died day 33 from MI, bronchopneumonia
Dineshkumar (2017)[17]	CS	2	India	D. russelii (1),	56M	Schistocytes, biopsy kidney	Yes	Yes	NR	AKI requiring dialysis	AV, TPE	NR	ESKD
				*Hypnale* spp. (1)	46F	Schistocytes, biopsy kidney	Yes	Yes	NR	AKI requiring dialysis	Nil	lost to follow up	Discharged against medical advice
Ehelepola (2019)[39]	CR	1	Sri Lanka	Hump nosed viper (*H*.*hypnale*)	47 F	Schistocytes	Yes	Yes	No. Minor bite site bleed	Dialysis requiring AKI; ? neurological (seizures, AION), ? cardiac (NSTEMI)	TPE	6 mo	Survival, ESKD, mild improvement in AION
Gn (2017)[15]	CR	1	India	NR	60 F	Schistocytes, biopsy: kidney	Yes	Yes	NR. Major bleed—GI, endo-tracheal	Dialysis requiring AKI	AV	NR	Survival, ESKD
Godavari (2016)[25]	CS	2	India	NR	30F, 49M	Schistocytes	Yes	Yes	NR (1), no (1)	AKI requiring dialysis	AV, TPE	20 d (1), NR (1)	DFS, renal function recovered
Harris (1976)[70]	CS	3	Australia	? Gwardar (*Demansia nuchalis nuchalis*)	35M	Schistocytes	Yes	Yes	NR	AKI requiring dialysis	-	3 y	DFS with normal renal function
				? Dugite (*Demansia nuchalis affinis*)	60M	Schistocytes	Yes	Yes	NR	AKI requiring dialysis	AV	1 y	DFS with partial renal recovery
				*Demansia nuchalis nuchalis*	53F	Schistocytes	Yes	Yes	Minor bleeding	AKI requiring dialysis	-	5.5 mo	DFS with near normal renal function
Hatten (2013)[40]	CR	1	USA	Great Lakes bush viper (Atheris *nitschei*)	30 M	Schistocytes	Yes	Yes	Complete VICC. Minor bleed–bite site, axillary haematoma, endotracheal	Nil	-	4.5 d	Survival, normal renal function
Herath (2012)[71]	CS	7	Sri Lanka	*Hypnale* spp.	70F	Schistocytes	Yes	Yes	WBCT >20min	AKI requiring dialysis, digital gangrene (1)	TPE	NR	DFS with recovered renal function, digital gangrene recovered (1)
					54F, 57M	Schistocytes	Yes	Yes	WBCT>20min	AKI requiring dialysis (2)	Nil	NR	DFS, renal function recovered (2)
					44M, 44F	Schistocytes	Yes	Yes	WBCT>20min	AKI requiring dialysis (2)	Nil		DFS, CKD (2)
					54F, 76F	Schistocytes, autopsy TMA in kidney, heart, spleen (2)	Yes	Yes	WBCT>20min	AKI requiring dialysis (2)	Nil	5 d	Death from refractory hypotension (2)
Joseph (2007)[72]	CS	2	India	*H*. *hypnale*	7M, 38M	Schistocytes	No (1), NR (1)	NR	Incoagulable blood	Nil	-	1.5–4 d	Survived, nil complications
Karthik (2004)[42]	CR	1	India	n/a	12 M	Schistocytes	Yes	Yes	Minor bleed	Dialysis requiring AKI	AV	28 d	DFS, normal renal function
Karunatilake (2012)[43]	CR	1	Sri Lanka	*Hypnale* spp.	35 M	Schistocytes	Yes	Yes	Not classifiable	Dialysis requiring AKI	-	Lost to follow up 3.5 d	Dialysis dependant at time of loss to follow up
Karunaranthne (2013)[44]	CR	1	Sri Lanka	*Hypnale* spp.	51 M	Schistocytes	Yes	Yes	Partial VICC	Dialysis requiring AKI with type IV renal tubular acidosis	-	26 w	DFS, renal recovery
Keyler (2008)[45]	CR	1	USA	Lowland viper (*Proatheris superciliaris*)	27 M	Schistocytes	Yes	Yes	Partial VICC. Minor bleed	AKI not requiring dialysis	TPE	1 y	Survival, normal renal function
Kularatne (2014)[46]	CR	1	Sri Lanka	Russell's viper (*Daboia russelii*)	43 F	Schistocytes	Yes	Yes	WBCT >20min. Minor bleed	AKI not requiring dialysis, ? brain (opthalmoplegia, bilateral ptosis)	AV	8 d	Survival, normal renal function
Mahasandana (1980)[83]	CS	1	Thailand	*D*. *russelii*	19M	Schistocytes	NR	Yes	NR. GI bleeding	AKI dialysis requirement NR	-	NR	NR
Malaque (2019)[73]	CS	2	Brazil	*B*. *jararaca*	70F, 71F	Schistocytes	Yes	Yes	Complete VICC	AKI requiring dialysis (1)	AV	8 w	DFS, normal renal function
										AKI not requiring dialysis (1)	AV	4 w	Survived, normal renal function
Malbranque (2008)[13]	CR	1	Martinique Is (Caribbean)	Fer-de-Lance pit viper (*B*. *lanceolatus*)	74 M	Schistocytes, autopsy: kidney, brain, heart, bowel	Yes	Yes	NR	Brain–infarcts, LOC and tetraplegia; cardiac–MI. No AKI (creatinine rise <1.5x normal),	AV	10 d	Death from left ventricular heart failure due to ruptured chordae tendinae
Mitrakrishnan (2012)[22]	CR	1	Sri Lanka	*H*. *hypnale*	70 M	Schistocytes	Yes	Yes	NR	Dialysis requiring AKI	TPE	NR	DFS, normal renal function
Namal (2018)[60]	CS	2†	Sri Lanka	*H*. *zara* (1), *H*. *nepa* (1)	53M, 70M	Schistocytes	Yes	No	No	Nil	-	6 h– 3 d	Survived, no complications
Namal (2019)[50]	CS	4	Sri Lanka	*H*. *hypnale* (3)	60F	Schistocytes	Yes	Yes	NR	AKI not requiring dialysis	-	7 d	Survived, renal function near normal
				*Hypnale spp*. (1)	74F, 57M, 55F	Schistocytes	Yes	Yes	1 NR, 1 c VICC, 1 p VICC	AKI requiring dialysis	TPE (3)	22 d, 4 mo	DFS (3), 1 recovered renal function, 1 ongoing ACD at 22 days (creatinine 241 mol/L), 1 Stage 4 CKD (eGFR 20ml/1.73m2) at 4 months
Namal (2017)[48]	CR	1	Sri Lanka	*D*. *russelii*	43 M	Schistocytes, autopsy: brain, spleen, lung	No	Yes	WBCT incoagulable. Major bleed—ICH	Brain–infarction. No AKI	AV	11 d	Death from cerebral haemorrhage and infarction, brain stem herniation
Namal (2017)[47]	CR	1	Sri Lanka	*H*. *hypnale*	74 F	schistocytes	Yes	No	NR. Minor m/c bleed	Nil	-	7 d	Survival
Namal (2019)[50]	CR	1	Sri Lanka	*D*. *russelii*	57 F	schistocytes	Yes	Yes	Partial VICC. Minor bleed—GI	Dialysis requiring AKI	AV	15 d	DFS, normal renal function
Namal (2018)[51]	CR	1	Sri Lanka	*H*. *hypnale*	42 M	Schistocytes; autopsy ? brain	Yes	Yes	Partial VICC	AKI not requiring dialysis, ? brain–small infarct	TPE	16 d	Death from multiorgan failure. Renal function normal at time of death
Namal (2020)[49]	CR	1	Sri Lanka	*H*. *zara*	65 M	Schistocytes	Yes	Yes	Partial VICC	AKI requiring dialysis	-	27 d	DFS, creatinine 525 μmol/L at time last follow up
Nicolson (1974)[52]	CR	1	UK	Boomslang/Sth African green tree snake (*Dispholidus typus*)	24 M	Schistocytes	Yes	Yes	Complete VICC. Minor bleed	AKI not requiring dialysis	AV	45 d	Survival, normal renal function
Rahmani (2020)[77]	CS	2	Israel	*E*. *coloratus*	39M, 70M	Schistocytes	Yes	Yes	Complete VICC	AKI requiring dialysis	AV, TPE (2)	2 mo (2)	DFS (2), 1 with normal renal function, 1 with DFS renal recovery otherwise NR
Satish (2017)[53]	CR	1	India	*D*. *russelii*	25 F pregnant	Schistocytes	Yes	Yes	VICC (p)	AKI requiring dialysis	AV	98 d	DFS, normal renal function, delivered baby premature labour 32 weeks
Schneemann (2004)[8]	CS	2	Switzerland (1)	Saharan horned viper (*Cerastes cerastes*)	63M	Schistocytes	Yes	Yes	Complete VICC, major bleed (ICH)	AKI requiring dialysis, ? heart (troponin elevation), rhabdomyolysis ? related	AV	20 mo	DFS, renal function normal
			UK (1)	*C*. *cerastes*	43M	Schistocytes	Yes	Yes	Partial VICC	AKI requiring dialysis	AV	37 d	DFS, renal function normal
Shastry (1977)[78]	CS	2	India	NR	25M, 35F	Biopsy; kidney	Yes	Yes	Partial VICC (1), NR (1). Bleed (2)	AKI requiring dialysis (2)	AV (1)	NR	Survival with CKD, with DFS NR (2)
Thillainathan (2015)[54]	CR	1	Sri Lanka	*H*. *hypnale*	49 M	Schistocytes	Yes	Yes	Partial VICC. Minor bleed	Cardiac, no AKI	-	6 mo	Survival, cardiac arrest, brain injury with severe cerebral disability at 6 months at last follow up
Uberoi (1991)[55]	CR	1	India	Viperidae	21 F	Biopsy: kidney	NR	Yes	Partial VICC. Minor bleed	Dialysis dependant AKI, delayed pan-hypopituitarism 2.5 y post snakebite, ? aetiology	AV	5 y	DFS, normal renal function, panhypopituitarism
Warrell (2009)[80]	CS	1	Seriname	Common lancehead pit viper (*B*. *atrox*)	37M	Schistocytes	Yes	Yes	Coagulopathy present (not classifiable), minor bleed	AKI requiring dialysis	AV	NR	DFS, normal renal function
Warrell (1975)[82]	CS	1	Nigeria	Puff adder (*Bitis arietans*)	18M	Schistocytes	Yes	No	Minor bleed	AKI dialysis requirement NR, ischaemic limb with popliteal artery thrombosis	-	24 d	Death from VF arrest, renal failure, paralytic ileus one day post limb amputation for limb gangrene
Weiss (1973)[56]	CR	1	USA	Saw scaled viper (*Echis carinatus*)	28 M	Schistocytes	NR	Yes	Complete VICC. Major bleed–GI, endotracheal	Nil	AV	15 d	Survival
White (1983)[57]	CR	1	Australia	Brown snake (*Pseudonaja nuchalis*)	26 M	Schistocytes	Yes	Yes	Partial VICC	Dialysis requiring AKI	AV	21 d	DFS, normal renal function
Withana 2014)[58]	CR	1	Sri Lanka	*H*. *hypnale*	55 F	Schistocytes	Yes	Yes	NR	AKI requiring dialysis, ? brain, ? cerebral	TPE	79 d	DFS, normal renal function
Zornig (2015)[59]	CR	1	Australia	Eastern Brown Snake (*P*. *textilis*)	45 M	Schistocytes. Minor bleed	Yes	Yes	Complete VICC	AKI requiring dialysis	AV	21 d	DFS

*Studies and cases tabulated after merging for case duplication.

†Case series reported 4 cases with partial duplication with PCS by same first author. 1 case merged with PCS and remaining 3 cases presented. TMA: thrombotic microangiopathy; Hb: haemoglobin; plt: platelets; CR: case report; VICC: venom induced consumption coagulopathy; AKI: acute kidney injury; AV: antivenom; DFS; dialysis free survival; d: days; CS: case series; NR: not reported; mo: months; WBCT: whole blood clotting time; TPE: plasmapheresis; w: weeks; MI: myocardial infarction; ESKD: end stage kidney disease; AION: acute ischaemic optic neuropathy; NSTEMI: non-ST elevation MI; y: years; LOC: loss of consciousness; ACD: acute kidney disease; CKD: chronic kidney disease; ICH: intracranial haemorrhage.

Four cases had ADAMTS-13 testing, and two cases complement C3 and C4 testing, all of which were normal [14,17,38,41,73,77]. One nested case control study found significantly increased red cell microvesicles in TMA compared to non-TMA snakebite cases; no significant difference in platelet microvesicles between TMA and non-TMA snakebite cases; and reduced endothelial microvesicles in all snakebite cases compared to normal controls [92].

GRADE assessment of strength of accumulated evidence was moderate for the outcome of AKI; and low to very low for DFS, other end organ damage and survival; attributable to the small observational study design, inconsistency and imprecision of results reporting of included studies (Tables 5 and S9).

**Table 5:** Quality of accumulated evidence by GRADE assessment.

No of cases*/studies†	Design	Risk of bias‡	Inconsistency	Indirectness	Imprecision	Other considerations	Quality of evidence
**Outcome: Proportion of cases with AKI**							
341/66	Observational studies	Not serious	Not serious	Not serious	Not serious	Very large magnitude of effect size	Moderate
**Outcome: Proportion of cases with other end organ damage**							
87/33	Observational studies	Serious (-1)	Very serious	Serious	Very serious	-	Very low
**Outcome: Survival**							
287/62	Observational studies	Not serious	Serious	Not serious	Serious	-	Very low
**Outcome: Proportion of patients with DFS–antivenom versus no antivenom**							
147/20	Observational studies	Not serious	Not serious	Serious	Serious	-	Very low
**Outcome: Proportion of patients with DFS–TPE versus no TPE**							
142/37	Observational studies	Not serious	Not serious	Not serious	Serious	Large magnitude of effect	Low

*Number of cases after merging.

†Number of studies represents all included studies of this systematic review.

‡Risk of bias assessed by framework from Murad et al [33]. Inconsistency, indirectness, imprecision and other considerations assessed as per checklist in Meader et al [93]. AKI: acute kidney injury; DFS: dialysis free survival; TPE: therapeutic plasmapheresis

## Discussion

We report the first systematic review to synthesise the reported prevalence, features and outcomes of TMA following snakebite. Snakebite associated TMA was reported in a broad range of different envenoming snake species and countries worldwide. Cases were predominantly from vipers in India, Sri Lanka followed by elapids from Australia, with smaller numbers from other countries. Previous studies have considered TMA in snakebite uncommon [17,22,38]. In the context of global estimates of 2.7 million snakebites and 81,000 to 138,000 deaths per annum globally attributable to snakebite, our 371 retrieved cases suggest snakebite associated TMA is a rare disease. However, we found TMA reported in 10–15% of Australian elapid envenomings, and 5.4% of proven and probable *Hypnale* bites in Sri Lanka, in studies with a low risk of selection bias. Consistent with these findings, a recent prospective cohort study from Sri Lanka published after our last database search date reported a prevalence of 11% TMA in a total of 103 proven *Hypnale* envenomings, applying our definition of TMA in this systematic review [94].

TMA following snakebite usually presented in association with coagulopathy, with a delayed thrombocytopenia and MAHA in the days post envenoming. Coagulation changes were typically of a VICC as evidenced by hypofibrinogenaemia and coagulation factor consumption marked by prolonged INR and APTT. Some studies reported less reliable coagulation abnormalities, such as prolonged WBCT. WBCT is a simple bedside test which measures the time to a clot forming within a whole blood sample when exposed to a foreign surface such as glass. Whole blood clotting times have limitations with respect to requirements for standardisation of glassware equipment and samples which can affect sensitivity and specificity in diagnosis of coagulopathy after snakebite. However, they remain in use globally as they are a simple bedside test available in resource limited settings [95–97]. Anaemia and thrombocytopenia were almost universally present, as seen in other TMAs such as HUS [98].

The predominant clinical organ injury was renal, with AKI occurring in over 90% of cases with TMA. The majority required dialysis for AKI over days to weeks, but most patients achieved DFS. Our finding of predominant renal end organ injury confirms similar findings in previous literature reviews [6,99]. Long term outcomes for renal recovery were heterogeneously reported, although most patients recovered partially or completely. Our findings on AKI prevalence and the spectrum of outcomes are potentially confounded by studies based in tertiary referral renal centres.

We found no evidence to support a beneficial effect of intervention with antivenom, although high quality studies with a low risk of selection bias, reporting of interventions and clearly ascertained clinical outcomes were few, and small in size. Snakebite associated TMA was found in studies reporting envenomings from *Hypnale*, for which no effective antivenom is available, but also reported in *D*. *russelii* and Australian elapids for which effective antivenom is available and was administered in almost every case. This made any estimate of treatment effect unreliable with very wide confidence intervals due the small numbers (often zero receiving antivenom, or conversely no cases of *Hypnale* receiving antivenom). In only one study did a reasonable proportion of patients not receive antivenom, but the information on antivenom was unreliable and few outcomes were reported [88]. This means that we found little evidence to support antivenom specifically in treatment of TMA following snakebite.

In addition, the clinical studies available provided little evidence as to whether timing of antivenom administration accounted for this lack of evident benefit of antivenom for TMA prevention. The prevention of envenoming seen in animal studies in which antivenom is administered before venom is injected, supports an hypothesis that for some manifestations of envenoming such as TMA, and potentially VICC, administration of antivenom following the bite to prevent that manifestation is so time-critical that in the vast majority of snakebite scenarios, antivenom is administered after the time-critical window [100–102]. Our findings do not detract from the critical role for antivenom in snakebite envenoming more broadly.

Similarly, we found no evidence to support a beneficial effect of intervention with TPE for renal outcomes in dialysis-dependant AKI complicating snakebite associated TMA. Cohort studies from Sri Lanka, India and Australia reporting outcomes for TPE intervention showed no statistically significant benefit for DFS. However, the quality of accumulated evidence in our review was low, predominantly owing to the small and observational included studies.

In contrast to our findings of predominant renal end organ injury, other end organ damage was uncommon, although our findings are limited by a risk of bias from included studies and uncertainty about TMA specifically with respect to causality. Clinical features of cardiac, cerebral, lung and pituitary involvement occurred in rare cases, but were not clearly attributable to TMA. Histological TMA was rarely found at autopsy in the heart, lung, pituitary gland and bowel, in studies of high or unclear risk of selection bias.

Of the small number of deaths, the majority were due to major haemorrhage, shock, refractory hypotension, or organ failure. It is likely the early haemorrhagic deaths occurred in relation to initial VICC.

The aetiology of TMA following snakebite remains unclear. ADAMTS-13 and complement testing were normal in the rare cases of TMA following snakebite which were tested. The pattern of end organ renal injury in TMA following snakebite is more like HUS than TTP, which tends to cause neurological changes. However, the tendency for renal recovery in TMA following snakebite differentiates it from complement mediated HUS, which tends to occur in adults with historically poor renal outcomes with a likelihood of end stage kidney disease and long term dialysis dependence [18–20].

The main limitations to this study include the quality of included studies, and limitations on pooling data between heterogenous studies. Included studies showed considerable heterogeneity with respect to study setting, design, selection and reporting. As hypothesised, most studies were small and single centre observational studies, predominantly case reports and case series. Settings varied considerably from cohort studies consisting of all enrolled snakebite envenomings, to more highly selected cohorts from renal referral centres, intensive care units, and renal pathology reporting centres. Consolidation and synthesis of data was limited by selection bias of included studies, and we synthesised our findings by stratifying studies according to this bias. This conferred a lower quality of accumulated evidence, and a resultant relatively weak strength of findings and any recommendations.

We propose that patients presenting with snakebite and VICC are at reasonable risk of developing a delayed TMA, with a predilection for renal involvement. In patients presenting with AKI in snakebite it is likely that the majority have underlying TMA. We recommend, where resources allow, all patients presenting with snake envenoming and VICC undergo careful observation for anaemia and thrombocytopenia, serial blood film examination for schistocytes, careful monitoring of renal function and urine output, and judicious use of supportive care including dialysis as required.

We found no evidence in support of interventions beyond routine supportive care for the treatment of snakebite associated TMA. However, antivenom is the mainstay of therapy for snake envenoming and should always be given in this setting, although our study found no evidence of benefit specifically for renal outcomes in snakebite associated TMA. Whilst we have found no convincing evidence of a role for TPE, included studies were small and the strength of any recommendations regarding its use are weak. TPE is resource intense, requiring tertiary level hospital care, blood donation and transfusion services, specialised equipment, and staffing. Treatment strategies for snakebite and other neglected tropical diseases must ensure efficient use of limited resources. We recommend that TPE is not routinely used in TMA following snakebite outside of high-quality research studies, and highlight the need for large, good quality prospective studies of snakebite associated TMA.

## Supporting information

S1 Text Inclusion criteria for histological findings consistent with thrombotic microangiopathy. (PDF)

S1 Table PRISMA checklist. (PDF)

S2 Table Full search strategy for PubMed. (PDF)

S3 Table Definitions for unifying and categorising data. (PDF)

S4 Table Risk of bias evaluation tool for methodological quality of case reports and case series. (PDF)

S5 Table Characteristics of included studies. (PDF)

S6 Table Reviewer judgements about risk of bias for included studies. (PDF)

S7 Table Histology findings for included cases: Renal biopsies and autopsies. (PDF)

S8 Table Dialysis free survival for patients with AKI requiring dialysis, for studies with a low risk of selection bias reporting intervention with plasmapheresis. (PDF)

S9 Table Reviewer agreement—Cohen’s kappa statistic for selection of included studies and data extraction for outcomes and interventions. (PDF)

S1 Fig Lowest recorded platelet counts by study. (PDF)

S2 Fig Lowest recorded haemoglobin by study. (PDF)

S3 Fig Maximum recorded LDH by study. (PDF)

S4 Fig Time to maximum coagulopathy. (PDF)

S5 Fig Time to lowest recorded haemoglobin. (PDF)

S6 Fig Time to lowest recorded platelet count by study. (PDF)

S7 Fig Time to maximum recorded LDH. (PDF)
