# Supplementary material for: Snakebite associated thrombotic microangiopathy: a systematic review of clinical features, outcomes, and evidence for interventions including plasmapheresis
Source: PLoS Negl Trop Dis. 2020 Dec 8;14(12):e0008936. doi: 10.1371/journal.pntd.0008936 (PMC7748274; doi:10.1371/journal.pntd.0008936)
Supplement: S1 Text — (PDF) [file pntd.0008936.s001.pdf]

S1 Text. Inclusion criteria for histological findings consistent with thrombotic microangiopathy

- small and micro-vessel fibrin or platelet thrombosis or deposits [1-4]

- small vessel wall endothelial injury evidenced by the following changes:
    - endothelial swelling, splitting, disruption or proliferation [1-4]
    - hyalinosis or fibrinoid necrosis [4]
    - chronic changes of myointimal proliferation resulting in concentric rings of cells and matrix surrounding small vessels, sometimes described as onion skinning in renal biopsies [2, 4]
    - fibrinoid necrosis of vessels [4]

- specifically, for renal histology:
    - ischaemic changes in glomeruli with subendothelial swelling and capillary luminal narrowing [1, 2]
    - thickening in small arteries and arterioles with restriction of lumen, aneurysmal dilatation or proliferation of arterioles at hilus of glomerulus [1]
    - a double contour of glomerular basement membrane [1, 2]

- thrombi were not required for pathological diagnosis in the presence of above vessel wall injury changes [1]. A double contour glomerular membrane was accepted as evidence of TMA only in the presence of other TMA changes given its non-specificity in isolation [5].
-

References

1. Brocklebank V, Wood KM, Kavanagh D. Thrombotic microangiopathy and the kidney. Clin J of the Am Soc of Nephrol. 2018;13:300-17.
2. Lusco MA, Fogo AB, Najafian B, Alpers CE. AJKD Atlas of Renal Pathology: Thrombotic Microangiopathy. American Journal of Kidney Diseases. 2016;68(6):e33-e4. doi: https://doi.org/10.1053/j.ajkd.2016.10.006.
3. El-Bietar J, Warren M, Dandoy C, Myers KC, Lane A, Wallace G, et al. Histologic Features of Intestinal Thrombotic Microangiopathy in Pediatric and Young Adult Patients after Hematopoietic Stem Cell Transplantation. Biology of Blood and Marrow Transplantation. 2015;21(11):1994-2001. doi: https://doi.org/10.1016/j.bbmt.2015.06.016.
4. Scully M, Cataland S, Coppo P, de la Rubia J, Friedman KD, Kremer Hovinga J, et al. Consensus on the standardization of terminology in thrombotic thrombocytopenic purpura and related thrombotic microangiopathies. J Thromb Haemost. 2017;15(2):312-22. Epub 2016/11/22. doi: 10.1111/jth.13571. PubMed PMID: 27868334.
5. Fogo AB, Lusco MA, Najafian B, Alpers CE. AJKD Atlas of renal pathology: membranoproliferative glomerulonephritis. American Journal of Kidney Diseases. 66(3):e19-e20.
