# Supplementary material for: Snakebite associated thrombotic microangiopathy: a systematic review of clinical features, outcomes, and evidence for interventions including plasmapheresis
Source: PLoS Negl Trop Dis. 2020 Dec 8;14(12):e0008936. doi: 10.1371/journal.pntd.0008936 (PMC7748274; doi:10.1371/journal.pntd.0008936)
Supplement: S2 Table — (PDF) [file pntd.0008936.s003.pdf]

S2 Table. Full search strategy for PubMed

Search #	Search term<br>(controlled vocabulary MeSH term or free text words)
#1	snakes [MeSH Terms]
#2	snake bites [MeSH Terms]
#3	venoms [MeSH Terms]
#4	#1 OR #2 OR #3
#5	thrombotic microangiopathies [MeSH Terms]
#6	thrombosis [MeSH Terms]
#7	erythrocytes [MeSH Terms]
#8	schistocyt* [Text Words]
#9	anemia, hemolytic [MeSH Terms]
#10	hemolysis [MeSH Terms]
#11	"red cell" [Text Words]
#12	fragment* [Text Words]
#13	#11 AND #12
#14	kidney diseases [MeSH Terms]
#15	multiple organ failure [MeSH Terms]
#16	#5 OR #6 OR #7 OR #8 OR #9 OR #10 OR #13 OR #14 OR #15
#17	#4 AND #16

* truncated search term. MeSH: medical subject headings.
