# Supplementary material for: Snakebite associated thrombotic microangiopathy: a systematic review of clinical features, outcomes, and evidence for interventions including plasmapheresis
Source: PLoS Negl Trop Dis. 2020 Dec 8;14(12):e0008936. doi: 10.1371/journal.pntd.0008936 (PMC7748274; doi:10.1371/journal.pntd.0008936)
Supplement: S3 Table — (PDF) [file pntd.0008936.s004.pdf]

S3 Table. Definitions for unifying and categorising data

Study variable or outcome	Plan to unify or categorise data
**MAHA**	
Haemoglobin nadir	Lowest recorded haemoglobin in g/L
Time to haemoglobin nadir	Time to lowest recorded haemoglobin in g/L from snake bite (hours and minutes)
Platelet nadir	Lowest recorded platelet count (10^9/L)
Time to platelet nadir	Time to lowest recorded platelet count (10^9/L) from snake bite (hours and minutes)
Maximum LDH	Maximum LDH (U/L) and as ratio of ULN for reported reference range (times ULN)
Time to maximum LDH	Time to maximum LDH (U/L) from snake bite (hours and minutes)
**VICC**	
Complete VICC	Undetectable fibrinogen by Clauss method; OR INR>3 and D-dimer at least 10 times assay ULN or >2.5mg/L
Partial VICC	Low but detectable fibrinogen, OR raised D-dimer AND maximum INR<3.0
**ISTH bleeding classification for non-surgical patients**	
Major	Fatal bleeding; or symptomatic bleeding in a critical area or organ (intracranial, intraspinal, intraocular, retroperitoneal, intra-articular, pericardial, or intramuscular with compartment syndrome); or bleeding causing fall in haemoglobin ≥20g/L, or leading to transfusion ≥2 units whole blood or red cells
Minor	All non-major bleeds
**KDIGO AKI stage***	
Stage 1	Day 1-7: creatinine 1.5-1.9 times baseline or ≥26.5 µmol/L increase; OR urine output <0.5ml/kg/h for 6-12 hours
Stage 2	Day 1-7: creatinine 2.0-2.9 times baseline; OR urine output <0.5ml/kg/h for ≥12 hours
Stage 3	Day 1-7: creatinine 3.0 times baseline or increase to ≥353.6 µmol/L; OR initiation of RRT; OR urine output <0.3ml/kg/h for ≥24 hours or anuria for ≥12 hours; OR in patients <18 years old, decrease in eGFR to <35ml/min/1.73m^2
**ADQI ACD stage†**	
Stage 1	Continuation of AKI stage 1 between day 8-90
Stage 2	Continuation of AKI stage 2 between day 8-90
Stage 3	Continuation of AKI stage 3 between day 8-90
**CKD stage**	
Stage 1	eGFR ≥90ml/min/1.73m^2 AND albuminuria, haematuria, a pathological abnormality or a structural abnormality
Stage 2	eGFR 60-89ml/min/1.73m^2
Stage 3a	eGFR 45-59ml/min/1.73m^2
Stage 3b	eGFR 30-44ml/min/1.73m^2
Stage 4	eGFR 15-29ml/min/1.73m^2
Stage 5	eGFR <15ml/min/1.73m^2

*KDIGO AKI staging is by most concerning feature of either creatinine rise, urine output or use of renal replacement therapy, during day 1 to 7 from renal insult. †ADQI ACD staging is defined as ongoing AKI between day 8 to 90. MAHA: microangiopathic haemolytic anaemia; LDH: lactate dehydrogenase; VICC: venom induced consumption coagulopathy; INR: International normalised ratio; ULN: upper limit of normal; KDIGO: Kidney Disease: Improving Global Outcomes; AKI: acute kidney injury; ADQI: Acute Disease Quality Initiative; ACD: acute kidney disease; CKD: chronic kidney disease; eGFR: estimated glomerular filtration rate
