# Supplementary material for: Snakebite associated thrombotic microangiopathy: a systematic review of clinical features, outcomes, and evidence for interventions including plasmapheresis
Source: PLoS Negl Trop Dis. 2020 Dec 8;14(12):e0008936. doi: 10.1371/journal.pntd.0008936 (PMC7748274; doi:10.1371/journal.pntd.0008936)
Supplement: S4 Table — (PDF) [file pntd.0008936.s005.pdf]

S4 Table. Risk of bias evaluation tool for methodological quality of case reports and case series

Derived from Murad et al [1]

Domain*	Relevant leading explanatory questions*
Selection	1. Does the study selection method of the case(s) enable a representation of the whole experience of the investigator or reporting centre or is the selection method unclear to the extent that other patients with similar presentation may not have been reported?
Ascertainment	2. Was the exposure of snakebite envenoming adequately ascertained?<br>3. Was (were) the outcome(s) adequately ascertained?
Causality	4. Were alternative causes that may explain the observation(s) ruled out?<br>5. Was follow up long enough for outcomes to occur?
Reporting	6. Is the case described with sufficient detail to allow other practitioners to make inferences relating to their own practice?

1. Murad MH, Sultan S, Haffar S, F B. Methodological quality and synthesis of case series and case reports. BMJ Evid-Based Med. 2018;23(2):60-3.
