# Supplementary material for: Snakebite associated thrombotic microangiopathy: a systematic review of clinical features, outcomes, and evidence for interventions including plasmapheresis
Source: PLoS Negl Trop Dis. 2020 Dec 8;14(12):e0008936. doi: 10.1371/journal.pntd.0008936 (PMC7748274; doi:10.1371/journal.pntd.0008936)
Supplement: S5 Table — (PDF) [file pntd.0008936.s006.pdf]

S5 Table. Characteristics of included studies

Study	Study design	Country	n	Snake (n)
Acharya 1989 [1]	CS	India	11	Viper
Ahlstrom 1991 [2]	CR	United States	1	Pigmy rattlesnake
Al Qahtani 2014 [3]	CR	Saudi Arabia	1	-
*Allen 2012 [4]	Multi centre PCS	Australia	15	Brown snake (*Pseudonaja* spp)
Amaral 1985 [5]	CS	Brazil	6	*Bothrops jararaca* (4), *B. jararacussu* (1), *Bothrops* spp. (1)
Aung 1978 [6]	CS	Burma	7	Russells viper (*Daboia russelii*)
Basu 1977 [7]	CS	India	9	*D. russelii*
Benvenuti 2003 [8]	CR	Brazil	1	*Bothrops jararacussu*
Bucaretchi 2019 [9]	CR	Brazil	1	*Bothrops jararaca*
*Casamento 2011 [10]	CS	Australia	2	Tiger snake *Notechis scutatus*
Chugh 1989 [11]	CS	India	2	Viper (1), Saw-scaled viper (1)
†Chugh 1984 [12]	CS	India	5	*D. russelii* (2), unknown (3)
†Chugh 1975 [13]	CS	India	2	*D. russelii* (2)
Cobcroft 1997 [14]	CR	Australia	1	Taipan *Oxyuranus scutellatus*
‡Date 1986 [15]	CS	India	22	*D. russelii* (15)
‡Date 1982 [16]	CS	India	9	*D. russelii* (9)
‡Date 1981 [17]	CS	India	2	*D. russelii* (2)
de Silva 2017 [18]	CR	Sri Lanka	1	Hump nosed viper (*Hypnale*)
Dineshkumar 2017 [19]	CS	India	2	D. *russelii* (1), Hump nosed viper (1)
Ehelepola 2019 [20]	CR	Sri Lanka	1	*Hypnale hypnale*
*Enjeti 2018 [21]	Nested CCS	Australia	13	*Pseudonaja* spp (8), unknown (5)
Gn 2017 [22]	CR	India	1	Unknown
Godavari 2016 [23]	CS	India	2	Unknown
Gupta 1988 [24]	CS	India	1	Unknown
Harris 1976 [25]	CS	Australia	3	Gwardar *D.nuchalis* (2), ? Dugite *D.nuchalis affinis*) (1)
Hatten 2013 [26]	CR	United States	1	Great Lakes Bush Viper (*Atheris nitschei*)
Herath 2012 [27]	CS	Sri Lanka	7	Hump nosed viper (*Hypnale* sp)
*Ho 2010 [28]	CR	Australia	1	Tiger snake *N. scutatus*
*Isbister 2007 [29]	Multi centre PCS and CS	Australia	6	Western Brown Snake *P. nuchalis*
*Johnston 2017 [30]	Multi centre PCS	Australia	6	Taipan (*Oxyuranus* spp)
Joseph 2007 [31]	CS	India	2	Hump-nosed viper (*Hypnale hypnale)*
Karthik 2004 [32]	CR	India	1	Unknown
Karunatilake 2012 [33]	CR	Sri Lanka	1	*H. hypnale*
Karunaranthne 2013 [34]	CR	Sri Lanka	1	*H. hypnale*
Keyler 2008 [35]	CR	USA	1	Lowland viper *P. superciliaris*
Kularatne 2014 [36]	CR	Sri Lanka	1	*D. russelii*
Mahasandana 1980 [37]	CS	Thailand	1	*D. russelii*
Malaque 2019 [38]	CS	Brazil	2	*Bothrops jararaca*
Malbranque 2008 [39]	CR	Caribbean	1	Fer-de-Lance pit viper B. *lanceolatus*
Merchant 1989 [40]	CS	India	11	NR
Milani Junior 1997 [41]	Single centre RCS and PCS	Brazil	2	B. *jararacussu*
Mitrakrishnan 2012 [42]	CR	Sri Lanka	1	Merrem's hump nosed viper
§Mittal 1994 [43]	CS	India	25	Viperidae
§Mittal 1986 [44]	CS	India	21	Viperidae
¶Mohan 2019 [45]	Single centre RCS	India	49	*D. russelii* (1), NR (48)
¶Mohan 2019 [46]	CR	India	1	*D. russelii* (1)
Namal 2019 [47]	CS	Sri Lanka	4	*Hypnale* spp (1), H. *hypnale* (3)
‖Namal 2018 [48]	CS	Sri Lanka	2	*H. zara* (1), *H. nepa* (1)

S5 Table. continued

Study	Study design	Country	n	Snake (n)
Namal 2017 [49]	CR	Sri Lanka	1	*D. russelii*
Namal 2017 [50]	CR	Sri Lanka	1	*H. hypnale*
Namal 2020 [51]	CR	Sri Lanka	1	*H. zara*
Namal 2019 [52]	CR	Sri Lanka	1	*D. russelii*
Namal 2018 [53]	CR	Sri Lanka	1	*H. hypnale*
‖Namal 2019 [54]	Single centre PCS	Sri Lanka	29	*H. hypnale* (12), *Hypnale* spp (17)
Nicolson 1974 [55]	CR	UK	1	Boomslang/Sth African green tree snake *D. typus*
*Noutsos 2012 [56]	CS	Australia	31	Brown snake (*Pseudonaja* spp.) (23), Taipan (*Oxyuranus* spp) (3), Tiger (*Notechis scutatus*) or tiger group (3), unknown (2)
Rahmani 2020 [57]	CS	Israel	2	Echis *coloratus*
Rao 2019 [58]	Single centre RCS	India	19	NR
Satish 2017 [59]	CR	India	1	*D. russelii*
Schneemann 2004 [60]	CS	UK and Switzerland	2	Saharan horned viper *C. cerastes*
Shastry 1977 [61]	CS	India	2	Unknown
Than-Than 1989 [62]	CS	Burma	2	*D. russelii*
Thillainathan 2015 [63]	CR	Sri Lanka	1	*H. hypnale*
Uberoi 1991 [64]	CR	India	1	Viperine
Warrell 1975 [65]	CS	Nigeria	1	Puff-adder *B.arietans*
Warrell 1977 [66]	Single centre RCS	Nigeria	8	E.*carinatus*
Warrell 2009 [67]	CS	Seriname	1	Common lancehead pit viper (*Bothrops atrox*)
Weiss 1973 [68]	CR	USA	1	*E. carinatus*
White 1983 [69]	CR	Australia	1	*P.nuchalis*
Wijewickrama 2020 [70]	Single centre PCS	Sri Lanka	47	Daboia (10), *Hypnale* (18), unknown (17), NR (2)
Withana 2014 [71]	CR	Sri Lanka	1	*H. hypnale*
Zornig 2015 [72]	CR	Australia	1	Eastern Brown *P. textilis*

*Studies with Australian Snakebite Project cases merged due to partial or complete case overlap. †‡§¶Studies with same first author merged due to partial or complete case overlap. CS: case series; CR: case report; PCS: prospective cohort study; CCS: case control study; RCS: retrospective cohort study; NR: not reported.

References

1. Acharya VN, Khanna UB, Almeida AF, Merchant MR. Acute Renal Failure Due to Viperine Snake Bite as Seen in Tropical Western India. Renal Failure. 1989;11(1):33-5. doi: 10.3109/08860228909066944.
2. Ahlstrom NG, Luginbuhl W, Tisher CC. Acute anuric renal failure after pigmy rattlesnake bite. Southern Medical Journal. 1991;84(6):783-5. PubMed PMID: 2052975.
3. Al Qahtani M, Altheaby A, Al Anazi T, Al Saad K, Binsalih S, Al Helail M. Snake bite complicated by acute kidney injury secondary to necrotizing glomerulonephritis. Saudi Journal of Kidney Diseases and Transplantation. 2014;25(6):1259-62. doi: 10.4103/1319-2442.144263.
4. Allen GE, Brown SGA, Buckley NA, O'Leary MA, Page CB, Currie BJ, et al. Clinical effects and antivenom dosing in brown snake (Pseudonaja spp.) envenoming--Australian snakebite project (ASP-14). Plos One. 2012;7(12):e53188-e. doi: 10.1371/journal.pone.0053188. PubMed PMID: 23300888.
5. Amaral CF, Da Silva OA, Goody P, Miranda D. Renal cortical necrosis following Bothrops jararaca and B. jararacussu snake bite. Toxicon. 1985;23(6):877-85. Epub 1985/01/01. doi: 10.1016/0041-0101(85)90379-4. PubMed PMID: 4095703.
6. Aung-Khin M. Histological and ultrastructural changes of the kidney in renal failure after viper envenomation. Toxicon. 1978;16(1):71-5. doi: https://doi.org/10.1016/0041-0101(78)90062-4.
7. Basu J, Majumdar G, Dutta A, al e. Acute renal failure following snake bite (viper). J Assoc Phys India. 1977;25:883-90.
8. Benvenuti LA, França FOS, Barbaro KC, Nunes JR, Cardoso JLC. Pulmonary haemorrhage causing rapid death after Bothrops jararacussu snakebite: a case report. Toxicon: Official Journal Of The International Society On Toxinology. 2003;42(3):331-4. PubMed PMID: 14559086.

9. Bucaretchi F, Pimenta MMB, Borrasca-Fernandes CF, Prado CC, Capitani EMD, Hyslop S. Thrombotic microangiopathy following Bothrops jararaca snakebite: case report. Clinical Toxicology. 2019;57(4):294-9. doi: 10.1080/15563650.2018.1514621.
10. Casamento AJ, Isbister GK. Thrombotic microangiopathy in two tiger snake envenomations. Anaesthesia And Intensive Care. 2011;39(6):1124-7. PubMed PMID: 22165369.
11. Chugh KS. Snake-bite-induced acute renal failure in India. Kidney International. 1989;35(3):891-907. PubMed PMID: 2651763.
12. Chugh KS, Pal Y, Chakravarty RN. Acute renal failure following poisonous snake bite. Am J Kidney Dis. 1984;4:30-8.
13. Chugh KS, Aikat BK, Sharma BK, Dash SC, Mathew MT, Das KC. Acute renal failure following snakebite. Am J Trop Med Hyg. 1975;24:692-7.
14. Cobcroft RG, Williams A, Cook D, Williams DJ, Masci P. Hemolytic uremic syndrome following taipan envenomation with response to plasmapheresis. Pathology. 1997;29(4):399-402. PubMed PMID: 9423222.
15. Date A, Pulimood R, Jacob CK, Kirubakaran MG, Shastry JCM. Haemolytic-uraemic syndrome complicating snake bite. Nephron. 1986;42:89-90.
16. Date A, Shastry JCM. Renal ultrastructure in acute tubular necrosis following Russell's viper envenomation. J Pathology. 1982;137:225-41.
17. Date A, Shastry JCM. Renal ulstrastructure in cortical necrosis following Russell's viper envenomation. Journal of Tropical Medicine and Hygiene. 1981;84:3-8.
18. de Silva NL, Gooneratne L, Wijewickrama E. Acute myocardial infarction associated with thrombotic microangiopathy following a hump-nosed viper bite: a case report. Journal Of Medical Case Reports. 2017;11(1):305. doi: 10.1186/s13256-017-1484-z. PubMed PMID: 29082854.
19. Dineshkumar T, Dhanapriya J, Sakthirajan R, Thirumalvalavan K, Kurien AA, Balasubramaniyan T, et al. Thrombotic microangiopathy due to Viperidae bite: Two case reports. Indian J Nephrol. 2017;27(2):161-4.
20. Ehelepola NDB, Karunathilaka CN, Liyanage GLHS, Wickramaarachchi WACB, Samarathunga JRPU, Dissanayake WP. An atypical clinical manifestation of a hump-nosed pit viper envenomation. Case Reports in Medicine. 2019;2019(Article ID 4172395). doi: https://doi.org/10.1155/2019/4172395.
21. Enjeti AK, Lincz LF, Seldon M, Isbister GK. Circulating microvesicles in snakebite patients with microangiopathy. Research and Practice in Thrombosis and Haemostasis. 2019;3(1):121-5. doi: 10.1002/rth2.12164.
22. Gn YM, Ponnusamy A, Thimma V. Snakebite induced thrombotic microangiopathy leading to renal cortical necrosis. Case Reports in Nephrology. 2017:10.1155/2017/1348749. doi: 10.1155/2017/1348749.
23. Godavari KSV. Hemolytic uremic syndrome - An unusual complication of snake envenomation. University Journal of Medicine and Medical Sciences, The Tamil Nadu Dr MGR Medical University. 2016;2(2).
24. Gupta A, Rao PV. Acute renal failure following snake bite. J Assoc Physicians India. 1988;36(8):501-3. Epub 1988/08/01. PubMed PMID: 3246503.
25. Harris AR, Hurst PE, Saker BM. Renal failure after snake bite. The Medical Journal Of Australia. 1976;2(11):409-11. PubMed PMID: 994917.
26. Hatten BW, Bueso A, French LK, Hendrickson RG, Horowitz BZ. Envenomation by the Great Lakes Bush Viper (Atheris nitschei). Clinical Toxicology (Philadelphia, Pa). 2013;51(2):114-6. doi: 10.3109/15563650.2012.763134. PubMed PMID: 23327286.
27. Herath N, Wazil A, Kularatne S. Thrombotic microangiopathy and acute kidney injury in hump-nosed viper (Hypnale species) envenoming: A descriptive study in Sri Lanka. Toxicon. 2012;60:61-5.
28. Ho WK, Verner E, Dauer R, Duggan J. ADAMTS-13 activity, microangiopathic haemolytic anaemia and thrombocytopenia following snake bite envenomation. Pathology. 2010;42(2):200-2. doi: 10.3109/00313020903493955. PubMed PMID: 20085531.
29. Isbister GK, Little M, Cull G, McCoubrie D, Lawton P, Szabo F, et al. Thrombotic microangiopathy from Australian brown snake (Pseudonaja) envenoming. Intern Med J. 2007;37(8):523-8. PubMed PMID: 17640187.
30. Johnston CI, Ryan NM, O'Leary MA, Brown SGA, Isbister GK. Australian taipan (Oxyuranus spp.) envenoming: clinical effects and potential benefits of early antivenom therapy - Australian Snakebite Project (ASP-25). Clinical Toxicology (Philadelphia, Pa). 2017;55(2):115-22. doi: 10.1080/15563650.2016.1250903. PubMed PMID: 27903075.
31. Joseph JK, Simpson ID, Menon NC, Jose MP, Kulkarni KJ, Raghavendra GB, et al. First authenticated cases of life-threatening envenoming by the hump-nosed pit viper (Hypnale hypnale) in India. Trans R Soc Trop Med Hyg. 2007;101(1):85-90. Epub 2006/07/15. doi: 10.1016/j.trstmh.2006.03.008. PubMed PMID: 16839578.
32. Karthik S, Phadke KD. Snakebite-induced acute renal failure. A case report and review of the literature. Pediatric Nephrology (Berlin, Germany). 2004;19(9):1053-4. PubMed PMID: 15179568.

33. Karunatilake H, Nayakarathna T, Atapattu S, Saparamadu T, Dharmasena S. Thrombotic microangiopathy and fibrinolysis after hump-nosed viper envenomation. The Ceylon Medical Journal. 2012;57(1):45-6. doi: 10.4038/cmj.v57i1.4204. PubMed PMID: 22453713.
34. Karunarathne S, Udayakumara Y, Govindapala D, Fernando H. Type IV renal tubular acidosis following resolution of acute kidney injury and disseminated intravascular coagulation due to hump-nosed viper bite. Indian J Nephrol. 2013;23:294-6.
35. Keyler DE. Envenomation by the lowland viper (Proatheris superciliaris): severe case profile documentation. Toxicon. 2008;52(8):836-41. Epub 2008/10/28. doi: 10.1016/j.toxicon.2008.10.013. PubMed PMID: 18950654.
36. Kularatne S, Wimalasooriya S, Nazar K, Maduwage K. Thrombotic microangiopathy following Russell's viper (Daboia russelii) envenoming in Sri Lanka: a case report. Ceylon Medical Journal. 2014;59:29-30.
37. Mahasandana S, Rungruxsirivorn Y, Chantarangkul V. Clinical manifestations of bleeding following Russell's viper and Green pit viper bites in adults. The Southeast Asian Journal Of Tropical Medicine And Public Health. 1980;11(2):285-93. PubMed PMID: 7434080.
38. Malaque CMS, Duayer IF, Santoro ML. Acute kidney injury induced by thrombotic microangiopathy in two cases of Bothrops envenomation. Clinical toxicology (Philadelphia, Pa). 2019;57(3):213-6. Epub 2018/11/15. doi: 10.1080/15563650.2018.1510129. PubMed PMID: 30430871.
39. Malbranque S, Piercecchi-Marti MD, Thomas L, Barbey C, Courcier D, Bucher B, et al. Case report: fatal diffuse thrombotic microangiopathy after a bite by the “fer-de-lance” pit viper (Bothrops lanceolatus) of Martinique. Am J Trop Med Hyg. 2008;78(6):856–61.
40. Merchant MR, Khanna UB, Almeida AF, Acharya VN, Mittal BV. Clinicopathological study of acute renal failure following viperine snake bite. The Journal Of The Association Of Physicians Of India. 1989;37(7):430-3.
41. Milani Júnior R, Jorge MT, de Campos FP, Martins FP, Bousso A, Cardoso JL, et al. Snake bites by the jararacuçu (Bothrops jararacussu): clinicopathological studies of 29 proven cases in São Paulo State, Brazil. QJM: An International Journal of Medicine. 1997;90(5):323-34. doi: 10.1093/qjmed/90.5.323.
42. Mitrakrishnan JY, Bandula CW, Mitrakrishnan CS, Somaratna K, Jeyalakshmy S. Haemolytic uremic syndrome a hitherto unreported complcation of humpnosed viper envenomation. Indian J of Hematol Blood Transfus. 2012;29(2):116-8.
43. Mittal BV. Acute renal failure following poisonous snake bite. J Postgrad Med. 1994;40:123.
44. Mittal BV, Kinare SG, Acharya VN. Renal lesions following viper bites - a study of 14 years. Indian J Med Res. 1986;83:642-51.
45. Mohan G, Guduri PR, Shastry S, Kandasamy D. Thrombotic microangiopathy in hematotoxic snakebites and its impact on the prognosis: an entity often overlooked. Journal of thrombosis and thrombolysis. 2019;48(3):475-82. doi: 10.1007/s11239-019-01868-z. PubMed PMID: 31028512.
46. Mohan G, Guduri PR, Shastry S. Role of therapeutic plasma exchange in snake bite associated thrombotic microangiopathy-A case report with review of literature. Journal of clinical apheresis. 2019;34(4):507-9. Epub 2019/02/19. doi: 10.1002/jca.21691. PubMed PMID: 30779435.
47. Namal Rathnayaka R, Ranathunga PAN, Kularatne SA. Thrombotic Microangiopathy, Hemolytic Uremic Syndrome, and Thrombotic Thrombocytopenic Purpura Following Hump-nosed Pit Viper (Genus: Hypnale) Envenoming in Sri Lanka. Wilderness & environmental medicine. 2019;30(1):66-78. Epub 2019/01/31. doi: 10.1016/j.wem.2018.10.003. PubMed PMID: 30711421.
48. Namal Rathnayaka RMMK, Ranathunga AN, Kularatne SAM, Rajapakse J, Ranasinghe S, Jayathunga R. Microangiopathic Hemolytic Anemia Following Three Different Species of Hump-Nosed Pit Viper (Genus Hypnale) Envenoming in Sri Lanka. Wilderness & Environmental Medicine. 2018;29(1):94-101. doi: 10.1016/j.wem.2017.11.003.
49. Namal Rathnayaka RMMK, Kularatne SAM, Kumarasinghe KDM, Ranaweera J, Nishanthi Ranathunga PEA. Ischemic brain infarcts and intracranial haemorrhages following Russell's viper (Daboia russelii) bite in Sri Lanka. Toxicon. 2017;125:70-3. doi: https://doi.org/10.1016/j.toxicon.2016.11.253.
50. Namal Rathnayaka RMMK, Kularatne SAM, Ranathunga AN, Kumarasinghe M, Rajapakse J, Ranasinghe S. Prolonged Coagulopathy, Ecchymoses, and Microangiopathic Hemolytic Anemia Following Hump-Nosed Pit Viper (Hypnale hypnale) Bite in Sri Lanka. Wilderness & Environmental Medicine. 2017;28(3):253-8. doi: 10.1016/j.wem.2017.05.005. PubMed PMID: 28755820.
51. Namal Rathnayaka RMMK, Nishanthi Ranathunga PEA, Kularatne SAM. Thrombotic Microangiopathy Following Hypnale zara (Hump-Nosed Pit Viper) Envenoming: The First Known Case Report from Sri Lanka. Wilderness & Environmental Medicine. 2020;31(1):71-7. doi: 10.1016/j.wem.2019.08.009.
52. Namal Rathnayaka RMMK, Nishanthi Ranathunga PEA. Thrombotic microangiopathy and hemolytic uremic syndrome following Russell's viper (Daboia russelii) bite. Ratnapura Clinical Society Journal. 2019:23-32.

53. Namal Rathnayaka R, Nishanthi Ranathunga PEA, Ranaweera J, Jayasekara K, Kularatne SAM. Cardiac arrest and atrial fibrillation in a patient after hump-nosed pit viper (Hypnale hypnale) bite. Toxicon. 2018;148:33-9. Epub 2018/04/03. doi: 10.1016/j.toxicon.2018.03.014. PubMed PMID: 29608921.
54. Namal Rathnayaka RMMK, Ranathunga PEAN, Kularatne SAM. Kidney injury following envenoming by hump-nosed pit viper (Genus: Hypnale) in Sri Lanka: proven and probable cases. Transactions of the Royal Society of Tropical Medicine and Hygiene. 2019;113(3):131-42. doi: 10.1093/trstmh/try120. PubMed PMID: 30544230.
55. Nicolson IC, Ashby PA, Johnson ND, Versey J, Slater L. Boomslang bite with haemorrhage and activation of complement by the alternate pathway. Clinical And Experimental Immunology. 1974;16(2):295-300. PubMed PMID: 4469217.
56. Noutsos T, Currie B, Brown SG, Isbister GK, editors. Thrombotic microangiopathy due to snake envenomation: a large case series. HAA-APSTH Annual Scientific Meeting (Haematology Society of Australia and New Zealand & Australian and New Zealand Society of Blood Transfusion & Australasian Society of Thrombosis and Haemostasis & Asia Pacific Society of Thrombosis and Haemostasis); 2012; Melbourne, Victoria, Australia.
57. Rahmani T-RI, Nasasra T, Fuchs L, Almog Y, Lurie Y, Galante O. Acute thrombotic microangiopathic kidney injury due to Echis coloratus envenomation. Clinical Toxicology. 2020;58(4):284-6. doi: 10.1080/15563650.2019.1629450.
58. Rao IR, Prabhu AR, Nagaraju SP, Rangaswamy D. Thrombotic Microangiopathy: An Under-Recognised Cause of Snake-bite-related Acute Kidney Injury. Indian journal of nephrology. 2019;29(5):324-8. doi: 10.4103/ijn.IJN_280_18. PubMed PMID: 31571738.
59. Satish H, Jayachandran S, Priyamvada PS, Keepanasseril A, Stravankumar J, Sankar G, et al. Snakebite-induced acute kidney injury requiring dialysis in second trimester of pregnancy: successful outcomes in a therapeutic quagmire. Saudi journal of kidney diseases and transplantation : an official publication of the Saudi Center for Organ Transplantation, Saudi Arabia. 2017;28(2):437-40.
60. Schneemann M, Cathomas R, Laidlaw ST, El Nahas AM, Theakston RD, Warrell DA. Life-threatening envenoming by the Saharan horned viper (Cerastes cerastes) causing micro-angiopathic haemolysis, coagulopathy and acute renal failure: clinical cases and review. QJM : monthly journal of the Association of Physicians. 2004;97(11):717-27. Epub 2004/10/22. doi: 10.1093/qjmed/hch118. PubMed PMID: 15496528.
61. Shastry JCM, Date A, Carman RH, Johny KV. Renal failure following snake bite. A clinicopathological study of nineteen patients. Am J Trop Med Hygeine. 1977;26:1032-8.
62. Than-Than., Francis N, Tin Nu S, Myint L, Tun P, Soe-Soe, et al. Contribution of focal haemorrhage and microvascular fibrin deposition to fatal envenoming by Russell's viper (Vipera russelli siamensis) in Burma. Acta Trop. 1989;46:23-38.
63. Thillainathan S, Priyangika D, Marasinghe I, Kanapathippillai K, Premawansa G. Rare cardiac sequelae of a hump-nosed viper bite. BMC Research Notes. 2015;8:437-. doi: 10.1186/s13104-015-1426-z. PubMed PMID: 26369415.
64. Uberoi HS, Achuthan AC, Kasthuri AS, Kolhe VS, Rao KR, Dugal JS. Hypopituitarism following snake bite. The Journal Of The Association Of Physicians Of India. 1991;39(7):579-80. PubMed PMID: 1800512.
65. Warrell DA, Ormerod LD, Davidson NM. Bites by puff-adder (Bitis arietans) in Nigeria, and value of antivenom. British Medical Journal. 1975;4(5998):697-700. PubMed PMID: 1203728.
66. Warrell DA, Davidson N, Greenwood BM, Ormerod LD, Pope HM, Watkins BJ, et al. Poisoning by bites of the saw-scaled or carpet viper (Echis carinatus) in Nigeria. The Quarterly Journal Of Medicine. 1977;46(181):33-62. PubMed PMID: 866568.
67. Warrell DA. Commissioned article: management of exotic snakebites. QJM: An International Journal of Medicine. 2009;102(9):593-601. doi: 10.1093/qjmed/hcp075.
68. Weiss HJ, Phillips LL, Hopewell WS, Phillips G, Christy NP, Nitti JF. Heparin therapy in a patient bitten by a saw-scaled viper (Echis carinatus), a snake whose venom activates prothrombin. American Journal of Medicine. 1973;54:653-??
69. White J, Fassett R. Acute renal failure and coagulopathy after snakebite. Medical Journal of Australia. 1983;2:142-3.
70. Wijewickrama ES, Gooneratne LV, Gnanathasan A, Gawarammana I, Gunatilake M, Isbister GK. Thrombotic microangiopathy and acute kidney injury following Sri Lankan Daboia russelii and Hypnale species envenoming. Clinical Toxicology. 2020:1-7. doi: 10.1080/15563650.2020.1717509.
71. Withana M, Rodrigo C, Gnanathasan A, Gooneratne L. Presumptive thrombotic thrombocytopenic purpura following a hump-nosed viper (Hypnale hypnale) bite: a case report. J Venom Anim Toxins Incl Trop Dis. 2014;20:26.
72. Zornig B. Acute kidney injury secondary to Eastern brown snake ('Pseudonaja textilis') bite: a case study. Renal Society of Australasia Journal. 2015;11(2):56-61.
