# Supplementary material for: Snakebite associated thrombotic microangiopathy: a systematic review of clinical features, outcomes, and evidence for interventions including plasmapheresis
Source: PLoS Negl Trop Dis. 2020 Dec 8;14(12):e0008936. doi: 10.1371/journal.pntd.0008936 (PMC7748274; doi:10.1371/journal.pntd.0008936)
Supplement: S6 Table — (PDF) [file pntd.0008936.s007.pdf]

S6 Table. Reviewer judgements about risk of bias for included studies

Risk of bias tool (Murad et al)[1]

Author	Year	Study design	Total study duration (recruitment)	Selection	Ascertainment (exposure)	Ascertainment (AKI)	Ascertainment (survival)	Ascertainment (other end organ damage)	Ascertainment (DFS)	Causality	Reporting
Acharya	1989	case series	15 years	yes	yes	yes	unclear	no	no	no	poor
Ahlstrom	1991	case report	n/a	unclear	yes	yes	yes	yes	yes	unclear	complete
Al Qahtani	2014	case report	n/a	unclear	yes	yes	yes	yes	yes	unclear	complete
Allen	2012	multi-centre PCS	8 years	yes	yes	yes	yes	unclear	unclear	unclear	poor
Amaral	1985	case series	10 years	yes	yes	yes	yes	no	unclear	unclear	poor
Aung	1978	case series	n/a	no	yes	yes	yes	no	no	no	poor
Basu	1977	case series	n/a	no	yes	yes	yes	no	no	no	poor
Benvenuti	2003	case report	n/a	unclear	yes	no	yes	yes	yes	no	poor
Bucaretchi	2019	case report	n/a	no	yes	yes	yes	yes	yes	yes	complete
Casamento	2011	case series	n/a	unclear	yes	yes	yes	no	yes	yes	partial
Chugh	1989	case series	n/a	unclear	yes	yes	yes	no	yes	unclear	complete
Chugh	1984	case series	16 years (1964-1980)	yes	yes	yes	unclear	unclear	no	yes	poor
Chugh	1975	case series	9 years (1964-73)	yes	yes	yes	yes	no	unclear	unclear	complete
Cobcroft	1997	case report	n/a	unclear	yes	yes	yes	yes	yes	unclear	partial
Date	1986	case series	8 years	yes	yes	yes	yes	no	no	no	poor
Date	1981	case series	n/a	unclear	yes	yes	yes	no	no	no	partial
Date	1982	case series	n/a	unclear	yes	yes	yes	no	yes	no	partial
de Silva	2017	case report	n/a	unclear	yes	yes	yes	yes	yes	no	complete
Dineshkumar	2017	case series	n/a	unclear	yes	yes	yes	no	yes	unclear	partial
Ehelopola	2019	case report	n/a	no	yes	yes	yes	yes	yes	yes	complete
Enjeti	2019	nested CCS	n/a	no	yes	no	no	no	no	no	poor
Gn	2017	case report	n/a	unclear	yes	yes	yes	yes	yes	no	complete
Godavari	2016	case series	n/a	unclear	yes	yes	yes	unclear	yes	no	partial
Gupta	1988	case series	4 years (1978-1982)	yes	unclear	unclear	yes	no	no	no	partial
Harris	1976	case series	n/a	unclear	yes	yes	yes	unclear	yes	no	poor
Hatten	2013	case report	n/a	unclear	yes	yes	yes	unclear	yes	unclear	complete
Herath	2012	case series	1 year	unclear	yes	yes	yes	yes	yes	unclear	partial
Ho	2010	case report	n/a	unclear	yes	yes	yes	yes	yes	yes	complete

S6 Table continued

Author	Year	Study design	Total study duration (recruitment)	Selection	Ascertainment (exposure)	Ascertainment (AKI)	Ascertainment (survival)	Ascertainment (other end organ damage)	Ascertainment (DFS)	Causality	Reporting
Isbister	2007	multi centre PCS	4 years	yes	yes	yes	yes	yes	yes	unclear	complete
Johnston	2017	multi centre PCS	13 years (2003-2016)	yes	yes	yes	unclear	unclear	unclear	unclear	poor
Joseph	2007	case series	n/a	unclear	yes	yes	yes	unclear	yes	unclear	partial
Karthik	2003	case report	n/a	unclear	yes	yes	yes	yes	yes	unclear	complete
Karunatilake	2012	case report	n/a	unclear	yes	yes	unclear	unclear	unclear	unclear	partial
Karunaranthne	2013	case report	n/a	unclear	yes	yes	yes	yes	yes	yes	partial
Keyler	2008	case report	n/a	unclear	yes	yes	yes	yes	yes	yes	complete
Kularatne	2014	case report	n/a	unclear	yes	yes	yes	no	yes	no	complete
Mahasandana	1980	case series	2 years	unclear	yes	yes	yes	unclear	no	no	partial
Malaque	2019	case series	n/a	unclear	unclear	yes	yes	yes	yes	yes	complete
Malbranque	2008	case report	n/a	unclear	yes	yes	yes	yes	yes	no	complete
Merchant	1989	case series	8 years (1977-1985)	yes	unclear	yes	no	no	no	unclear	partial
Milani Junior	1997	single centre RCS and PCS	20 years	yes	yes	yes	yes	yes	yes	yes	complete
Mitrakrishnan	2012	case report	n/a	unclear	unclear	yes	yes	yes	yes	yes	partial
Mittal	1986	case series	14 years (1971-1984)	yes	yes	yes	yes	no	no	unclear	poor
Mittal	1994	case series	23 years (1971-1993)	yes	yes	yes	no	no	no	no	partial
Mohan	2019	case report	n/a	unclear	unclear	yes	yes	yes	yes	yes	complete
Mohan	2019	single centre RCS	3 years	yes	unclear	yes	yes	yes	no	no	poor
Namal	2019	case series	n/a	unclear	yes	yes	yes	yes	yes	unclear	complete
Namal	2018	case series	n/a	unclear	yes	yes	yes	yes	yes	yes	complete
Namal	2017	case report	n/a	no	yes	yes	yes	yes	yes	yes	complete
Namal	2017	case report	n/a	unclear	yes	yes	yes	unclear	yes	unclear	partial
Namal	2020	case report	n/a	no	yes	yes	yes	yes	yes	unclear	partial
Namal	2019	case report	n/a	no	yes	yes	yes	unclear	yes	unclear	partial
Namal	2018	case report	n/a	no	yes	yes	yes	yes	yes	unclear	complete
Namal	2019	single centre PCS	4 years (2014-2018)	yes	yes	yes	unclear	unclear	no	unclear	partial
Nicolson	1974	case report	n/a	unclear	yes	yes	yes	unclear	yes	yes	complete

S6 Table continued

Author	Year	Study design	Total study duration (recruitment)	Selection	Ascertainment (exposure)	Ascertainment (AKI)	Ascertainment (survival)	Ascertainment (other end organ damage)	Ascertainment (DFS)	Causality	Reporting
Noutsos	2012	case series	8 years (2004-2012)	yes	yes	yes	yes	unclear	yes	unclear	complete
Rahmani	2020	case series	n/a	unclear	yes	yes	yes	unclear	yes	yes	complete
Rao	2019	single centre RCS	6 years (2012 to 2017)	yes	yes	yes	yes	yes	yes	yes	partial
Satish	2017	case report	n/a	unclear	yes	yes	yes	unclear	yes	yes	complete
Schneemann	2004	case series	n/a	unclear	yes	yes	yes	yes	yes	unclear	complete
Shastry	1977	case series	n/a	unclear	yes	yes	unclear	unclear	unclear	unclear	poor
Than-Than	1989	case series	2 years	yes	yes	unclear	yes	yes	yes	unclear	partial
Thillainathan	2015	case report	n/a	unclear	yes	yes	yes	yes	yes	yes	partial
Uberoi	1991	case report	n/a	no	yes	yes	yes	yes	yes	unclear	partial
Warrell	1975	case series	3 years	unclear	yes	unclear	yes	yes	yes	unclear	poor
Warrell	1977	single centre RCS	3 years	yes	yes	unclear	unclear	unclear	unclear	no	partial
Warrell	2009	case series	n/a	unclear	yes	yes	yes	unclear	yes	no	partial
Weiss	1973	case report	n/a	unclear	yes	yes	yes	unclear	yes	yes	complete
White	1983	case report	n/a	unclear	yes	yes	yes	unclear	yes	unclear	complete
Wijewickrama	2020	single centre PCS	2 years	yes	yes	yes	yes	no	yes	unclear	complete
Withana	2014	case report	n/a	unclear	yes	yes	yes	yes	yes	yes	complete
Zornig	2015	case report	n/a	unclear	yes	yes	yes	yes	yes	yes	complete

AKI: acute kidney injury; DFS: dialysis free survival; PCS: prospective cohort study; RCS: retrospective cohort study; CCS: case control study

1. Murad MH, Sultan S, Haffar S, F B. Methodological quality and synthesis of case series and case reports. BMJ Evid-Based Med. 2018;23(2):60-3.
