# Supplementary material for: Snakebite associated thrombotic microangiopathy: a systematic review of clinical features, outcomes, and evidence for interventions including plasmapheresis
Source: PLoS Negl Trop Dis. 2020 Dec 8;14(12):e0008936. doi: 10.1371/journal.pntd.0008936 (PMC7748274; doi:10.1371/journal.pntd.0008936)
Supplement: S7 Table — (PDF) [file pntd.0008936.s008.pdf]

S7 Table. Histology findings for included cases: renal biopsies and autopsies

Study	Biopsy type	Site	Findings
Acharya [1]	biopsy and necropsy	kidney	7/29 fibrin thrombi and ACN on renal Bx (2) or autopsy (5), 10/29 ballooning capillaries (7 autopsy, 3 Bx), 19/29 splitting and thickening of GBM (8 autopsy, 11 Bx), 10/29 swelling of endothelial cells (6 autopsy, 4 biopsy)
Al Qahtani [2]	biopsy	kidney	necrotising GN, glom ischaemic change, segmental scarring with obliteration of glomerular capillaries, GBM segmental corrugation, prominent endothelial cells in glom capillaries, glom necrotic, mild arteriolar hyalinosis, consistent with acute TMA with segmental glomerular scarring
Amaral [3]	biopsy and necropsy	kidney	Renal cortical necrosis, coagulative necrosis of glomeruli, fibrin thrombi in capillary loops and small vessels
Aung [4]	necropsy	kidney	7 biopsies reported. Consistent findings were of renal cortical necrosis, glomerular coagulation, fibrin glomerular deposits, capillary loops distended and filled with masses of red blood cells, platelets and granular and amorphous fibrin masses. Most of the capillary luminae totally occluded. Electron microscopy showed masses and strands of fibrin, red blood cells and intervening areas packed with fibrin strands, disintegrated platelets and dense granular material. Deposited fibrin in large fibrillar and granular masses, thrombi, swelling of endothelial cell cytoplasm and some disruption of endothelium
Basu [5]	histology	kidney	Unclear reporting, ischaemic changes and isolated tuft glomerular fibrin thrombosis in some biopsies, fibrin thrombi in arterioles in other biopsies
Benvenuti [6]	necropsy	lung, heart	Extensive alveolar haemorrhage and oedema with numerous platelet thrombi in capillaries and venules. Myocardium showed foci of fibrosis and interstitial haemorrhage; multiple fibrin and platelet thrombi present in microcirculation. Immunohistochemical reactivity for factor VIII confirmed the presence of platelets in the pulmonary and myocardial thrombi
Chugh [7]	biopsy	kidney	ATN
	biopsy	kidney	ACN, fibrin thrombi, extensive glom and tubular necrosis
Chugh [8, 9]	biopsy and necropsy	kidney	In at least 1 case an autopsy showed cortical necrosis, fibrin thrombi in glom capillaries and arterioles and cortical necrosis.
Cobcroft [10]	necropsy	kidney, spleen, lung	Renal necropsy findings included a fibrin clot in interlobular artery, and scanty fibrin staining in glomeruli; fibrin thrombi also present in the small arteries of spleen, pulmonary arterioles. Infarcts in spleen and single small infarct in kidney. Cause of death: cerebral cortex hypoxic diffuse injury due to cardiac arrest. The brain and heart did not show signs of TMA on histology
Date [11-13]	biopsy	kidney	15 patients' renal biopsy: cortical necrosis in 3 cases and ATN in 12. Fibrin and platelet clusters were demonstrable in glomeruli and microvessels in 5 of 7 biopsies examined by electron microscopy. Some cases showed necrosis of peritubular capillaries with fragmentation of endothelial cells; irregular GBM thickening and wrinkling, focal endothelial swelling, occasional degenerating endothelial cells. Electron microscopy showed granular material and platelet clusters in vessel luminae, subendothelial deposits, blood vessel endothelial cell swelling, medullary blood vessels severely affected with marked swollen focally necrotic endothelial cells almost completely obliterating the lumen, occasional platelet clusters also seen. The three with cortical necrosis did not recover renal function. Those with ATN all recovered
Dineshkumar [14]	biopsy	kidney	More than 80% cortical necrosis with fibrin thrombi involving arteries and glomerular capillaries. Immunohistochemisty negative for immunoglobulin and complement

S7 Table continued

Study	Biopsy type	Site	Findings
Gn [15]	biopsy	kidney	Cortical necrosis with segmental necrosis and luminal thrombotic occlusion in the arteries and arterioles
Gupta [16]	necropsy	kidney	Fibrin thrombi in glomerular capillaries
Herath [17]	necropsy ~~(double check)~~	kidney, heart, spleen	2 necropsies with extensive TMA in small and medium sized arteries of kidneys, heart, spleen; multiple glomerular capillary thrombi and glomerular and tubular epithelial necrosis; microscopic foci of myocardial cell necrosis
Isbister [18]	biopsy	kidney	1 biopsy showing fibrin thrombi and red cell sludging in glomerular capillaries, some apoptotic cellular debris and possible early segmental necrosis
Karunaranthne [19]	biopsy	kidney	Subacute interstitial nephritis, no ATN but this is possibly due to late biopsy timing relative to snakebite and AKI
Malbranque [20]	necropsy	kidney, brain, heart, bowel	Multiple cerebral, myocardial and mesenteric infarctions and rupture of mitral valve chordae tendinae likely cause of death; multi-focal TMA with intimal-medial dissection by thrombi extending from foci of endothelial damage in small cerebral, myocardial, pulmonary, mesenteric, and interlobular renal arteries and arterioles, which were the cause of the infarctions; intense angiogenesis in organising cerebral infarcts; platelet aggregates and endothelial cells within microthrombi
Merchant [21]	necropsy	kidney	ACN (5 necropsies, 2 biopsies), fibrin thrombi in glomeruli; and at least 4 other cases non-ACN (2 of which were necropsies) showing capillary thrombi or ballooning, endothelial swelling
Milani Junior [22]	biopsy	kidney (1 biopsy); kidney, muscle, bowel, brain (1 necropsy)	Biopsy (1) showed diffuse cortical and medullary necrosis, proliferative GN, ATN, fibrin in walls of arterioles and fibrin thrombi in some glomerular capillaries. Necropsy (1) showed muscle and interstitial necrosis and DIC in region of wound, fibrin thrombi in small sub-mucosal vessels of intestine, with ischaemic necrosis of mucosa and in small cortical and meningeal vessels with local meningeal haemorrhage and cerebral oedema
Mittal [23]	biopsy (8), necropsy (13) - check.	kidney	Biopsies showed ACN or ATN, fresh and endothelialised glomerular thrombi, endothelial swelling, capillary ballooning, GBM thickening or splitting, regeneration of tubular necrosis; necropsies showed ACN or ATN changes, glomerular necrosis, glomerular thrombi, tubular necrosis, endothelial swelling, GBM splitting or thickening, capillary ballooning, congestion of vessels, fibrous intimal thickening
Namal [24]	necropsy	brain, spleen, lung	Haemorrhages, plus thrombi and congested blood vessels in cerebrum, spleen and lungs; kidneys and liver were normal
Namal [25]	necropsy	brain	Renal ATN, cardiac histology was normal, pulmonary haemorrhages, small brain infarct near basal ganglia
Namal [26]	biopsy	kidney	Sclerosed glomeruli, mesangial matrix expansion and hypercellularity, occasional glomeruli with fragmented red cells, some glomeruli with fibrin thrombi
Rao [27]	biopsy	kidney	2 biopsies; 1 with patchy cortical necrosis with fibrin thrombi in glomerular capillary lumens and arterioles (day 18), patient had full renal recovery; 1 (day 31) with patchy cortical necrosis, fibrin thrombi in glomerular capillary lumen, and evidence of AIN, patient remained dialysis dependant with ESKD

S7 Table continued

Study	Biopsy type	Site	Findings
Shastry [28]	biopsy	kidney	Cortical necrosis, fibrin in glomerular capillaries
Than-Than [29]	necropsy	kidney, pituitary, lung (1); pituitary, lung (1)	1 necropsy showed pituitary acute congestion and some fibrinous material in sinusoids, no haemorrhage; lungs showed fibrin thrombi in intra-septal capillaries and some larger pulmonary vessels; kidneys showed fibrin thrombi in glomerular tufts, and early ATN, renal arteries contained fresh thrombi; other organs were normal. 1 necropsy showed small thrombi in pituitary gland sinusoids; kidneys showed early tubular necrosis but no thrombi; even though patient had AKI; lungs had intense capillary wall congestion
Uberoi [30]	biopsy	kidney	ATN and microthrombi in vasculature

Bx: biopsy; ACN: acute cortical necrosis; GBM: glomerular basement membrane; GN: glomerulonephritis; TMA: thrombotic microangiopathy; ATN: acute tubular necrosis; DIC: disseminated intravascular coagulation; ESKD: end stage kidney disease

References

1. Acharya VN, Khanna UB, Almeida AF, Merchant MR. Acute Renal Failure Due to Viperine Snake Bite as Seen in Tropical Western India. Renal Failure. 1989;11(1):33-5. doi: 10.3109/08860228909066944.
2. Al Qahtani M, Altheaby A, Al Anazi T, Al Saad K, Binsalih S, Al Helail M. Snake bite complicated by acute kidney injury secondary to necrotizing glomerulonephritis. Saudi Journal of Kidney Diseases and Transplantation. 2014;25(6):1259-62. doi: 10.4103/1319-2442.144263.
3. Amaral CF, Da Silva OA, Goody P, Miranda D. Renal cortical necrosis following Bothrops jararaca and B. jararacussu snake bite. Toxicon. 1985;23(6):877-85. Epub 1985/01/01. doi: 10.1016/0041-0101(85)90379-4. PubMed PMID: 4095703.
4. Aung-Khin M. Histological and ultrastructural changes of the kidney in renal failure after viper envenomation. Toxicon. 1978;16(1):71-5. doi: https://doi.org/10.1016/0041-0101(78)90062-4.
5. Basu J, Majumdar G, Dutta A, al e. Acute renal failure following snake bite (viper). J Assoc Phys India. 1977;25:883-90.
6. Benvenuti LA, França FOS, Barbaro KC, Nunes JR, Cardoso JLC. Pulmonary haemorrhage causing rapid death after Bothrops jararacussu snakebite: a case report. Toxicon: Official Journal Of The International Society On Toxinology. 2003;42(3):331-4. PubMed PMID: 14559086.
7. Chugh KS. Snake-bite-induced acute renal failure in India. Kidney International. 1989;35(3):891-907. PubMed PMID: 2651763.
8. Chugh KS, Aikat BK, Sharma BK, Dash SC, Mathew MT, Das KC. Acute renal failure following snakebite. Am J Trop Med Hyg. 1975;24:692-7.
9. Chugh KS, Pal Y, Chakravarty RN. Acute renal failure following poisonous snake bite. Am J Kidney Dis. 1984;4:30-8.
10. Cobcroft RG, Williams A, Cook D, Williams DJ, Masci P. Hemolytic uremic syndrome following taipan envenomation with response to plasmapheresis. Pathology. 1997;29(4):399-402. PubMed PMID: 9423222.
11. Date A, Pulimood R, Jacob CK, Kirubakaran MG, Shastry JCM. Haemolytic-uraemic syndrome complicating snake bite. Nephron. 1986;42:89-90.
12. Date A, Shastry JCM. Renal ultrastructure in acute tubular necrosis following Russell's viper envenomation. J Pathology. 1982;137:225-41.
13. Date A, Shastry JCM. Renal ulstrastructure in cortical necrosis following Russell's viper envenomation. Journal of Tropical Medicine and Hygiene. 1981;84:3-8.

14. Dineshkumar T, Dhanapriya J, Sakthirajan R, Thirumalvalavan K, Kurien AA, Balasubramaniyan T, et al. Thrombotic microangiopathy due to Viperidae bite: Two case reports. Indian J Nephrol. 2017;27(2):161-4.
15. Gn YM, Ponnusamy A, Thimma V. Snakebite induced thrombotic microangiopathy leading to renal cortical necrosis. Case Reports in Nephrology. 2017:10.1155/2017/1348749. doi: 10.1155/2017/1348749.
16. Gupta A, Rao PV. Acute renal failure following snake bite. J Assoc Physicians India. 1988;36(8):501-3. Epub 1988/08/01. PubMed PMID: 3246503.
17. Herath N, Wazil A, Kularatne S. Thrombotic microangiopathy and acute kidney injury in hump-nosed viper (Hypnale species) envenoming: A descriptive study in Sri Lanka. Toxicon. 2012;60:61-5.
18. Isbister GK, Little M, Cull G, McCoubrie D, Lawton P, Szabo F, et al. Thrombotic microangiopathy from Australian brown snake (Pseudonaja) envenoming. Intern Med J. 2007;37(8):523-8. PubMed PMID: 17640187.
19. Karunarathne S, Udayakumara Y, Govindapala D, Fernando H. Type IV renal tubular acidosis following resolution of acute kidney injury and disseminated intravascular coagulation due to hump-nosed viper bite. Indian J Nephrol. 2013;23:294-6.
20. Malbranque S, Piercecchi-Marti MD, Thomas L, Barbey C, Courcier D, Bucher B, et al. Case report: fatal diffuse thrombotic microangiopathy after a bite by the "fer-de-lance" pit viper (Bothrops lanceolatus) of Martinique. Am J Trop Med Hyg. 2008;78(6):856–61.
21. Merchant MR, Khanna UB, Almeida AF, Acharya VN, Mittal BV. Clinicopathological study of acute renal failure following viperine snake bite. The Journal Of The Association Of Physicians Of India. 1989;37(7):430-3.
22. Milani Júnior R, Jorge MT, de Campos FP, Martins FP, Bousso A, Cardoso JL, et al. Snake bites by the jararacuçu (Bothrops jararacussu): clinicopathological studies of 29 proven cases in São Paulo State, Brazil. QJM: An International Journal of Medicine. 1997;90(5):323-34. doi: 10.1093/qjmed/90.5.323.
23. Mittal BV, Kinare SG, Acharya VN. Renal lesions following viper bites - a study of 14 years. Indian J Med Res. 1986;83:642-51.
24. Namal Rathnayaka RMMK, Kularatne SAM, Kumarasinghe KDM, Ranaweera J, Nishanthi Ranathunga PEA. Ischemic brain infarcts and intracranial haemorrhages following Russell's viper (Daboia russelii) bite in Sri Lanka. Toxicon. 2017;125:70-3. doi: https://doi.org/10.1016/j.toxicon.2016.11.253.
25. Namal Rathnayaka R, Nishanthi Ranathunga PEA, Ranaweera J, Jayasekara K, Kularatne SAM. Cardiac arrest and atrial fibrillation in a patient after hump-nosed pit viper (Hypnale hypnale) bite. Toxicon. 2018;148:33-9. Epub 2018/04/03. doi: 10.1016/j.toxicon.2018.03.014. PubMed PMID: 29608921.
26. Namal Rathnayaka RMMK, Ranathunga PEAN, Kularatne SAM. Kidney injury following envenoming by hump-nosed pit viper (Genus: Hypnale) in Sri Lanka: proven and probable cases. Transactions of the Royal Society of Tropical Medicine and Hygiene. 2019;113(3):131-42. doi: 10.1093/trstmh/try120. PubMed PMID: 30544230.
27. Rao IR, Prabhu AR, Nagaraju SP, Rangaswamy D. Thrombotic Microangiopathy: An Under-Recognised Cause of Snake-bite-related Acute Kidney Injury. Indian journal of nephrology. 2019;29(5):324-8. doi: 10.4103/ijn.IJN_280_18. PubMed PMID: 31571738.
28. Shastry JCM, Date A, Carman RH, Johny KV. Renal failure following snake bite. A clinicopathological study of nineteen patients. Am J Trop Med Hygeine. 1977;26:1032-8.
29. Than-Than., Francis N, Tin Nu S, Myint L, Tun P, Soe-Soe, et al. Contribution of focal haemorrhage and microvascular fibrin deposition to fatal envenoming by Russell's viper (Vipera russelli siamensis) in Burma. Acta Trop. 1989;46:23-38.
30. Uberoi HS, Achuthan AC, Kasthuri AS, Kolhe VS, Rao KR, Dugal JS. Hypopituitarism following snake bite. The Journal Of The Association Of Physicians Of India. 1991;39(7):579-80. PubMed PMID: 1800512.
