# Supplementary material for: Snakebite associated thrombotic microangiopathy: a systematic review of clinical features, outcomes, and evidence for interventions including plasmapheresis
Source: PLoS Negl Trop Dis. 2020 Dec 8;14(12):e0008936. doi: 10.1371/journal.pntd.0008936 (PMC7748274; doi:10.1371/journal.pntd.0008936)
Supplement: S8 Table — (PDF) [file pntd.0008936.s009.pdf]

S8 Table. Dialysis free survival for patients with AKI requiring dialysis, for studies with a low risk of selection bias reporting intervention with plasmapheresis

Outcome by study	TPE n/N (%)	No TPE n (%)	Calculated odds ratio	95% CI	p value
Australian Snakebite Project Studies (2012) [1-7]					
D-AKI	5	8	-	-	-
DFS	5/5 (100%)	8/8 (100%)	0.65	0.01-37.67	0.83
ESKD	0/5 (0%)	0/8 (0%)	1.55	0.03-89.96	0.83
Death	0/5 (0%)	0/8 (0%)	1.55	0.03-89.96	0.83
Rao (2019) [8]					
D-AKI	3	12	-	-	-
DFS	3/3 (100%)	9/12 (75%)	2.58	0.10-63.69	0.56
ESKD	0/3 (0%)	2/12 (17%)	0.60	0.02-15.76	0.76
Death	0/3 (0%)	1/12 (8%)	1.09	0.04-33.38	0.96
Wijerickrama (2020) [9]					
D-AKI	5	23	-	-	-
DFS	5/5 (100%)	21/23 (91%)	1.28	0.05-30.68	0.88
ESKD	0/5 (0%)	0/23 (0%)	4.27	0.08-240.01	0.48
Death	0/5 (0%)	2/23 (9%)	0.78	0.03-18.75	0.88

References

1. Noutsos T, Currie B, Brown SG, Isbister GK, editors. Thrombotic microangiopathy due to snake envenomation: a large case series. HAA-APSTH Annual Scientific Meeting (Haematology Society of Australia and New Zealand & Australian and New Zealand Society of Blood Transfusion & Australasian Society of Thrombosis and Haemostasis & Asia Pacific Society of Thrombosis and Haemostasis); 2012; Melbourne, Victoria, Australia.
2. Allen GE, Brown SGA, Buckley NA, O'Leary MA, Page CB, Currie BJ, et al. Clinical effects and antivenom dosing in brown snake (Pseudonaja spp.) envenoming--Australian snakebite project (ASP-14). Plos One. 2012;7(12):e53188-e. doi: 10.1371/journal.pone.0053188. PubMed PMID: 23300888.
3. Isbister GK, Little M, Cull G, McCoubrie D, Lawton P, Szabo F, et al. Thrombotic microangiopathy from Australian brown snake (Pseudonaja) envenoming. Intern Med J. 2007;37(8):523-8. PubMed PMID: 17640187.
4. Enjeti AK, Lincz LF, Seldon M, Isbister GK. Circulating microvesicles in snakebite patients with microangiopathy. Research and Practice in Thrombosis and Haemostasis. 2019;3(1):121-5. doi: 10.1002/rth2.12164.
5. Johnston CI, Ryan NM, O'Leary MA, Brown SGA, Isbister GK. Australian taipan (Oxyuranus spp.) envenoming: clinical effects and potential benefits of early antivenom therapy - Australian Snakebite Project (ASP-25). Clinical Toxicology (Philadelphia, Pa). 2017;55(2):115-22. doi: 10.1080/15563650.2016.1250903. PubMed PMID: 27903075.
6. Casamento AJ, Isbister GK. Thrombotic microangiopathy in two tiger snake envenomations. Anaesthesia And Intensive Care. 2011;39(6):1124-7. PubMed PMID: 22165369.

7. Ho WK, Verner E, Dauer R, Duggan J. ADAMTS-13 activity, microangiopathic haemolytic anaemia and thrombocytopenia following snake bite envenomation. Pathology. 2010;42(2):200-2. doi: 10.3109/00313020903493955. PubMed PMID: 20085531.
8. Rao IR, Prabhu AR, Nagaraju SP, Rangaswamy D. Thrombotic Microangiopathy: An Under-Recognised Cause of Snake-bite-related Acute Kidney Injury. Indian journal of nephrology. 2019;29(5):324-8. doi: 10.4103/ijn.IJN_280_18. PubMed PMID: 31571738.
9. Wijewickrama ES, Gooneratne LV, Gnanathasan A, Gawarammana I, Gunatilake M, Isbister GK. Thrombotic microangiopathy and acute kidney injury following Sri Lankan Daboia russelii and Hypnale species envenoming. Clinical Toxicology. 2020:1-7. doi: 10.1080/15563650.2020.1717509.
