# Supplementary material for: Snakebite associated thrombotic microangiopathy: a systematic review of clinical features, outcomes, and evidence for interventions including plasmapheresis
Source: PLoS Negl Trop Dis. 2020 Dec 8;14(12):e0008936. doi: 10.1371/journal.pntd.0008936 (PMC7748274; doi:10.1371/journal.pntd.0008936)
Supplement: S9 Table — (PDF) [file pntd.0008936.s010.pdf]

S9 Table. Reviewer agreement - Cohen's kappa statistic for selection of included studies and data extraction for outcomes and interventions

Variable	Cohen's kappa statistic	95% confidence interval
Study selection for systematic review	0.92	(0.86-0.97)
Data extraction – AKI outcome	0.90	(0.83-0.97)
Data extraction – DFS outcome	0.93	(0.87-0.96)
Data extraction – overall survival outcome	0.80	(0.75-0.86)
Data extraction – other organ damage outcome	0.71	(0.64-0.79)
Data extraction – plasmapheresis intervention	0.83	(0.77-0.88)
Data extraction – antivenom intervention	0.87	(0.82-0.92)

AKI: acute kidney injury; DFS: dialysis free survival
