# Supplementary figures and images for: Snakebite associated thrombotic microangiopathy: a systematic review of clinical features, outcomes, and evidence for interventions including plasmapheresis
Source: PLoS Negl Trop Dis. 2020 Dec 8;14(12):e0008936. doi: 10.1371/journal.pntd.0008936 (PMC7748274; doi:10.1371/journal.pntd.0008936)

**S1 Fig. Lowest recorded platelet counts by study**  
(median, range)

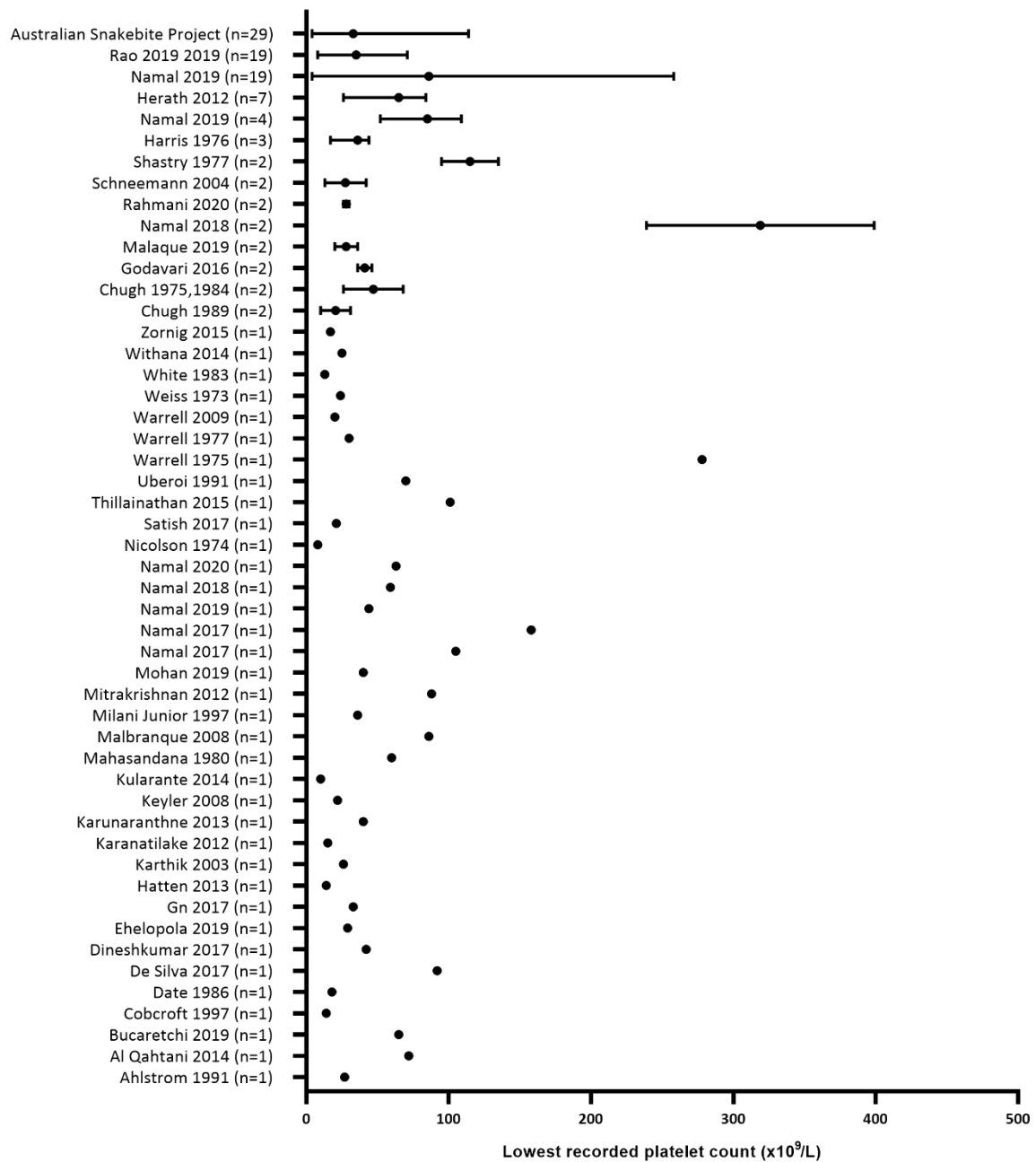

Supplement: S1 Fig — (PDF) [file pntd.0008936.s011.pdf]

**S2 Fig. Lowest recorded haemoglobin by study**  
(median, range)

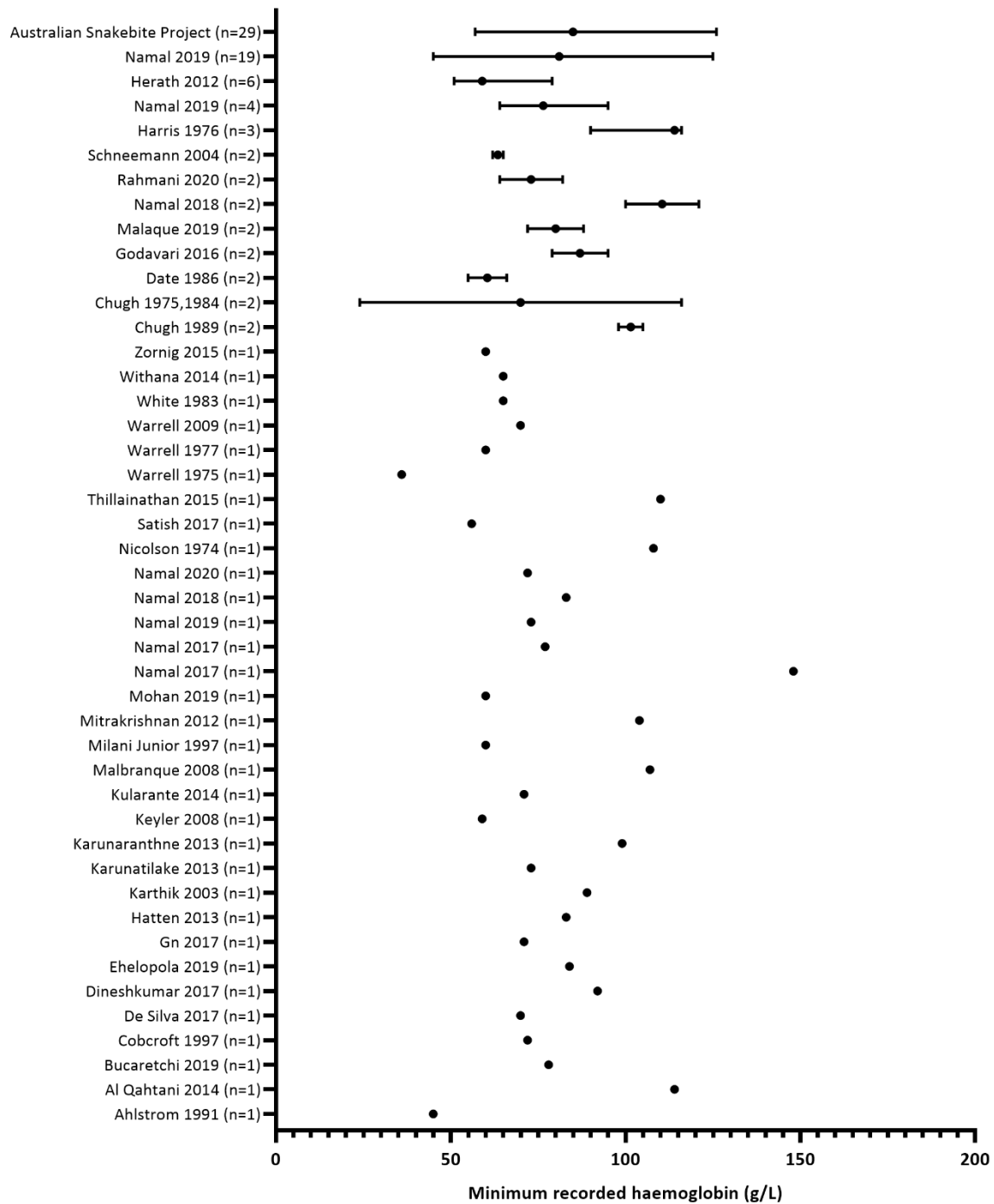

Supplement: S2 Fig — (PDF) [file pntd.0008936.s012.pdf]

**S3 Fig. Maximum recorded LDH by study**  
(median, range)

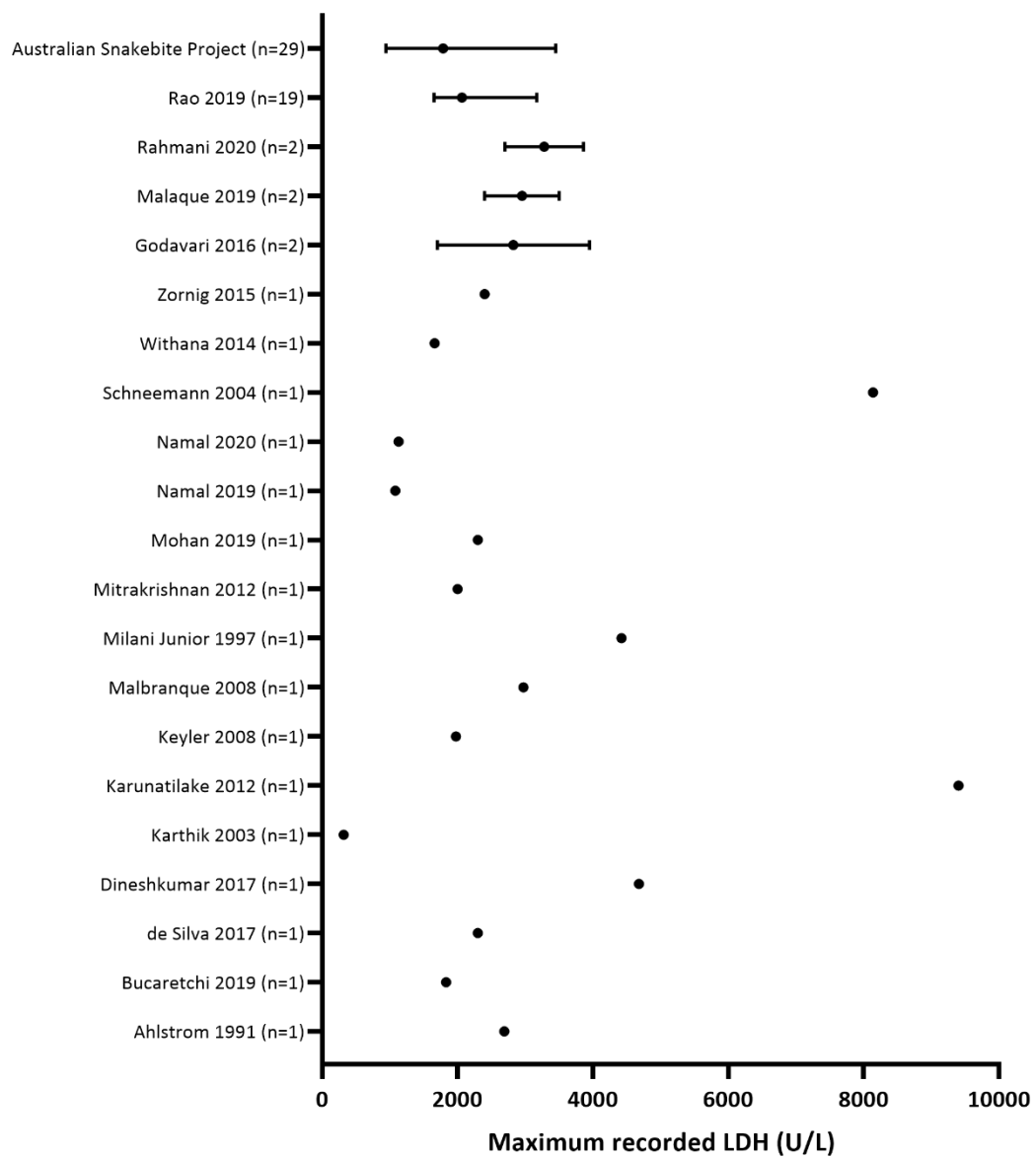

Supplement: S3 Fig — (PDF) [file pntd.0008936.s013.pdf]

**S4 Fig. Time to maximum coagulopathy**  
(median, range)

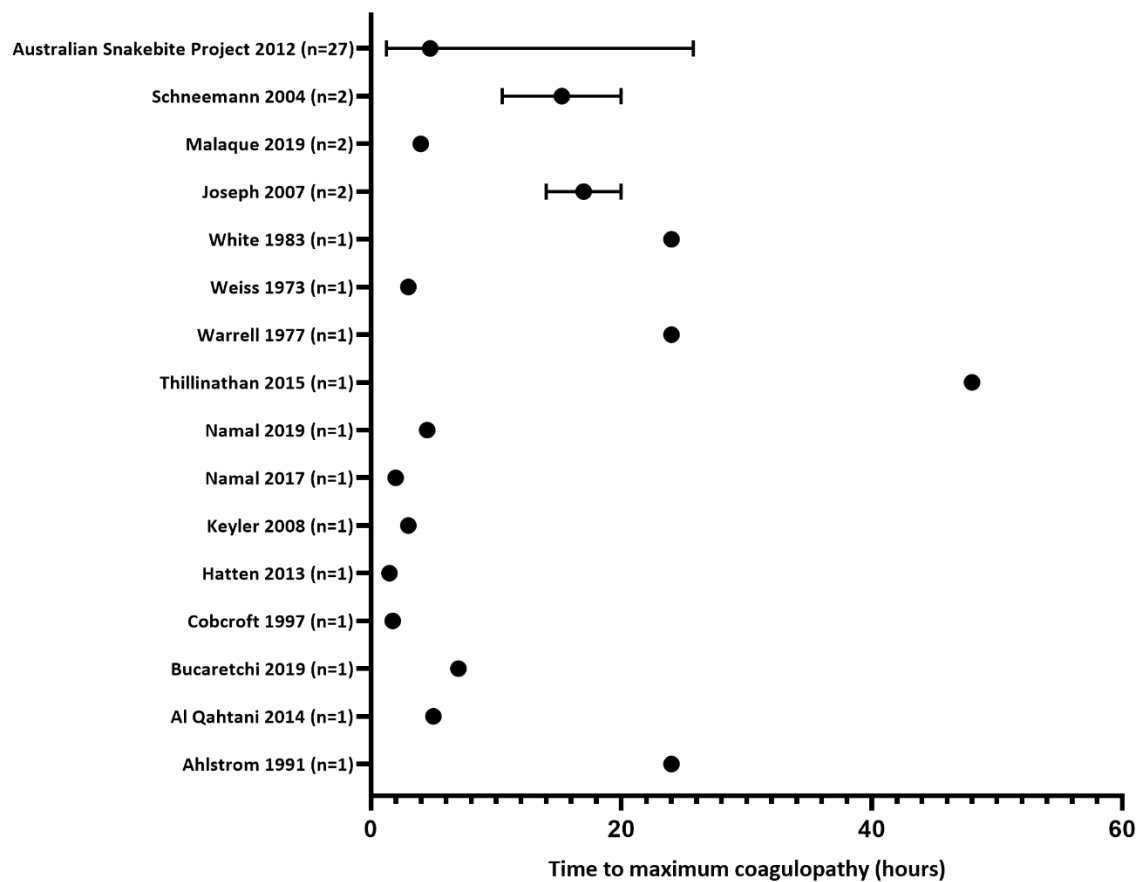

Supplement: S4 Fig — (PDF) [file pntd.0008936.s014.pdf]

**S5 Fig. Time to lowest recorded haemoglobin**  
(median, range)

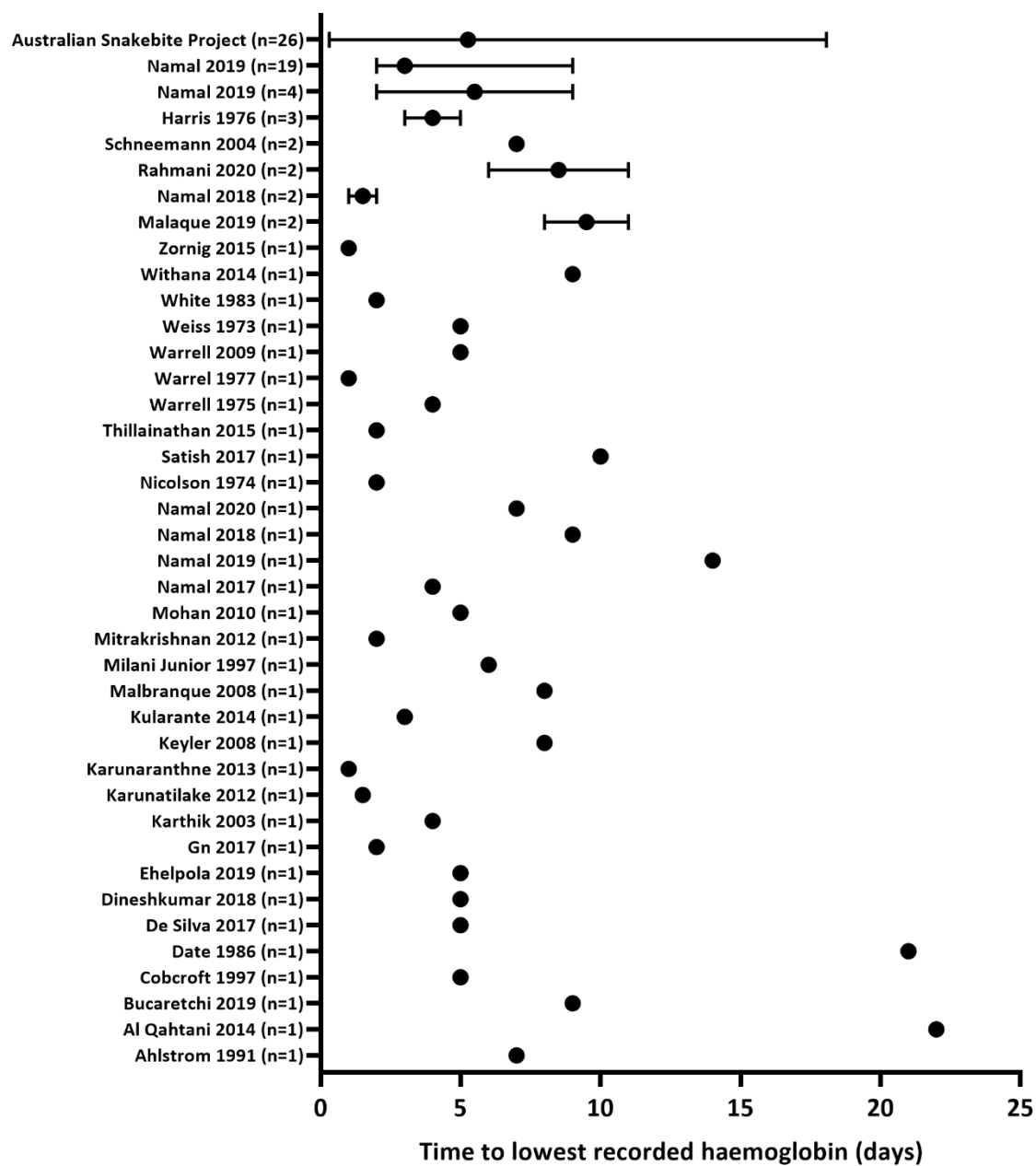

Supplement: S5 Fig — (PDF) [file pntd.0008936.s015.pdf]

**S6 Fig. Time to lowest recorded platelet count by study**  
(median, range)

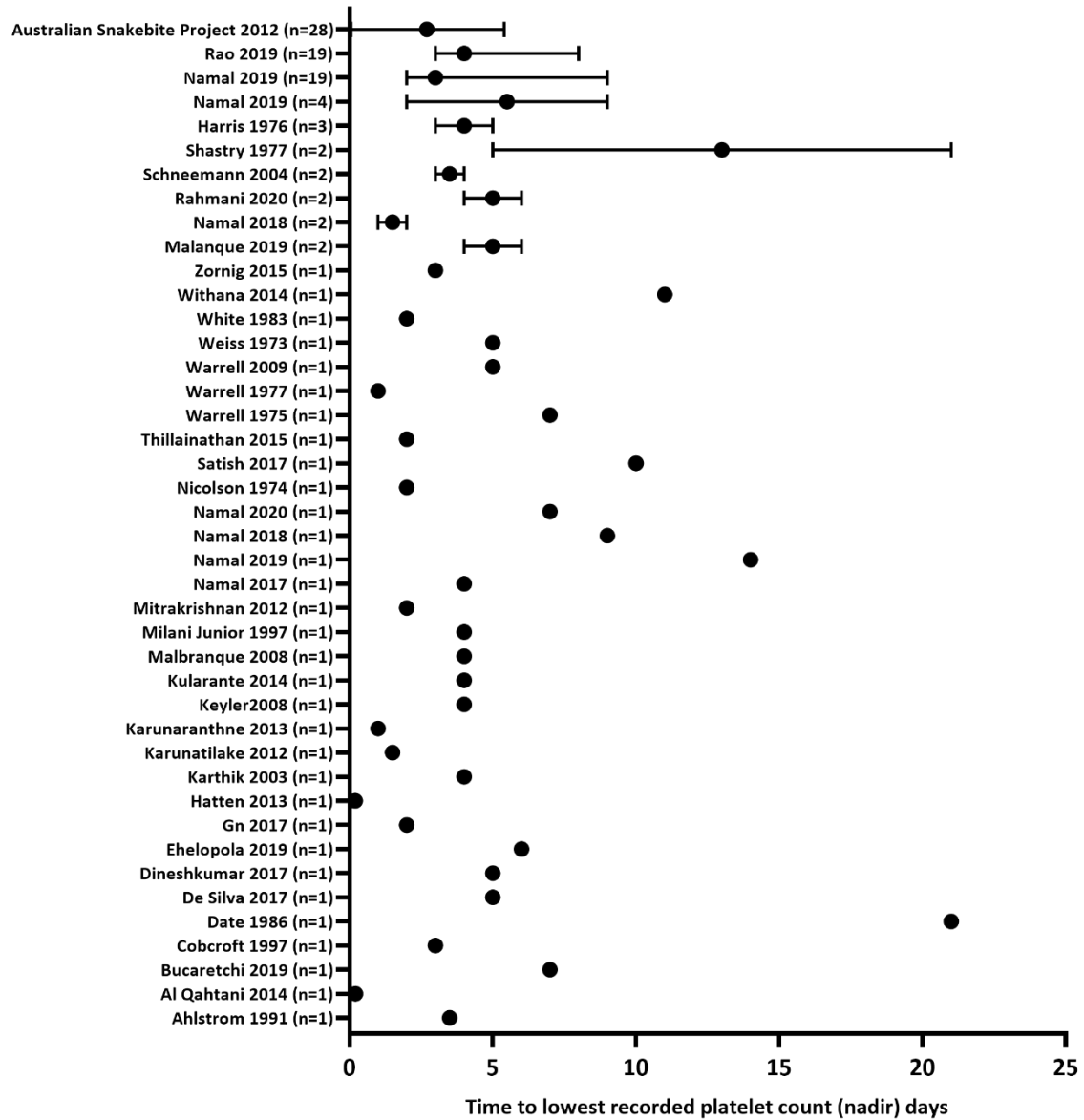

Supplement: S6 Fig — (PDF) [file pntd.0008936.s016.pdf]

**S7 Fig. Time to maximum recorded LDH**  
(median, range)

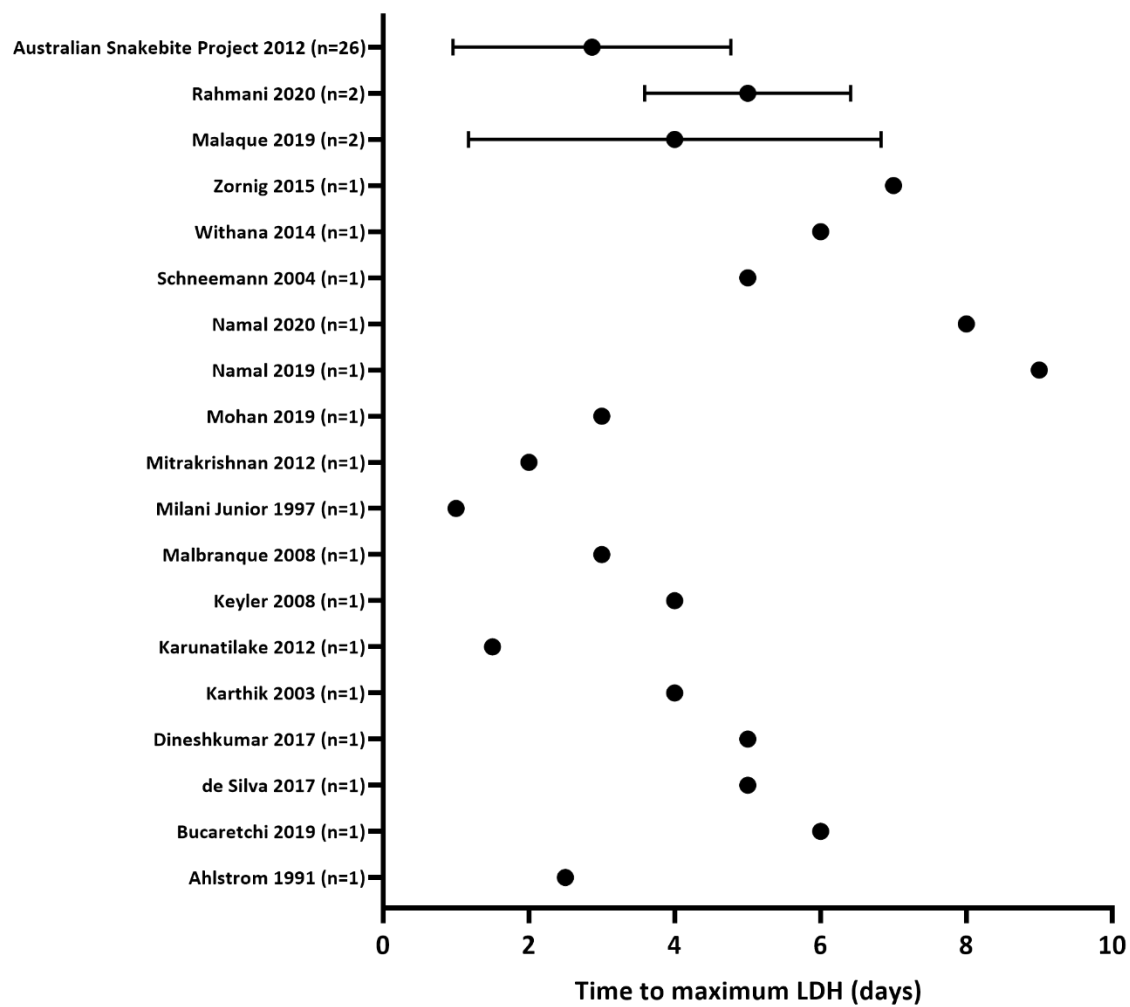

Supplement: S7 Fig — (PDF) [file pntd.0008936.s017.pdf]
